# CRISPR Typing and Subtyping for Improved Laboratory Surveillance of *Salmonella* Infections

**DOI:** 10.1371/journal.pone.0036995

**Published:** 2012-05-18

**Authors:** Laëtitia Fabre, Jian Zhang, Ghislaine Guigon, Simon Le Hello, Véronique Guibert, Marie Accou-Demartin, Saïana de Romans, Catherine Lim, Chrystelle Roux, Virginie Passet, Laure Diancourt, Martine Guibourdenche, Sylvie Issenhuth-Jeanjean, Mark Achtman, Sylvain Brisse, Christophe Sola, François-Xavier Weill

**Affiliations:** 1 Institut Pasteur, Unité des Bactéries Pathogènes Entériques, Paris, France; 2 Institute of Genetics and Microbiology, UMR8621, IGEPE Team, Universud, CNRS Université Paris-Sud 11, Orsay, France; 3 Institut Pasteur, Genotyping of Pathogens and Public Health, Paris, France; 4 Environmental Research Institute, University College Cork, Cork, Ireland; St. Petersburg Pasteur Institute, Russian Federation

## Abstract

Laboratory surveillance systems for salmonellosis should ideally be based on the rapid serotyping and subtyping of isolates. However, current typing methods are limited in both speed and precision. Using 783 strains and isolates belonging to 130 serotypes, we show here that a new family of DNA repeats named CRISPR (clustered regularly interspaced short palindromic repeats) is highly polymorphic in *Salmonella*. We found that CRISPR polymorphism was strongly correlated with both serotype and multilocus sequence type. Furthermore, spacer microevolution discriminated between subtypes within prevalent serotypes, making it possible to carry out typing and subtyping in a single step. We developed a high-throughput subtyping assay for the most prevalent serotype, Typhimurium. An open web-accessible database was set up, providing a serotype/spacer dictionary and an international tool for strain tracking based on this innovative, powerful typing and subtyping tool.

## Introduction

Salmonellosis is one of the most common causes of food-borne diarrheal disease worldwide. Most infections are zoonotic and are transmitted from food animals to humans through the ingestion of contaminated food. In the United States, 1.4 million nontyphoidal *Salmonella* infections are thought to occur in humans annually, resulting in approximately 15,000 hospitalizations and 400 deaths [1]. An efficient surveillance system for salmonellosis is therefore crucial. Various non exclusive strategies have been developed, including sentinel surveillance, periodic population-based surveys, and laboratory-based surveillance. Laboratory-based approaches are a key component of monitoring strategies in developed countries. They require a network of clinical laboratories covering the population and referring isolates or information to a central public health reference laboratory. The speed with which public health laboratories obtain information after the onset of symptoms and the regular sharing of information between public health laboratories and epidemiologists are critical for the successful use of information to detect outbreaks early and to identify their source. The basic information currently provided by laboratories is the serotype of the isolates. Hence, each year, more than 200,000 human isolates of *Salmonella* are serotyped in the United States and Europe [2], [3]. Serotyping, the reference method for *Salmonella* typing since the 1930s, is based on the determination of two surface antigens– O-polysaccharide and flagellin proteins – by agglutination with a large set of polyclonal rabbit antisera. This technique can recognize more than 2,500 serotypes [4], but its discriminatory capacity is limited, because two serotypes, Typhimurium and Enteritidis, are highly prevalent worldwide and account for most outbreaks. The sensitivity of serotyping for the detection of outbreaks involving these common serotypes, even with the use of cluster-detection algorithms, is therefore unsatisfactory [5].

Differentiation between isolates within the most common serotypes requires the use of subtyping methods, which were initially based on determination of the sensitivity of certain *Salmonella* serotypes to several bacteriophage suspensions (phage typing) [6]. DNA-based subtyping methods were subsequently developed, including, in particular, pulsed-field gel electrophoresis (PFGE) [7], which is based on analysis of the restriction pattern of high-molecular weight DNA digested with a rare-cutting restriction enzyme. Real-time subtyping methods have increased the power of laboratory-based surveillance to detect outbreaks, distinguishing them from the background of sporadic cases by identifying the phage type or molecular “fingerprint” of an outbreak strain. PFGE is currently the gold standard method for this purpose. This real-time subtype surveillance has been implemented in the US through PulseNet, an internet-based network of public health and food regulatory agency laboratories that perform real-time standardized PFGE and submit normalized PFGE patterns or raw TIFF gel images electronically to a national database. Regular searches of this database are made, with a view to identifying clusters of identical patterns. However, PFGE has several limitations: it is a technically demanding, non automated method. This may explain why, in a study of outbreaks of food-infection occurring in the US in 2002, the median interval from the onset of symptoms to PFGE results was 18, with a period of 10 days elapsing between the submission of isolates to public health laboratories and PFGE results [8]. Furthermore, the interpretation and comparison of banding profiles is not straightforward, even with standard protocols and analysis software. The discovery of short DNA sequence repeats in the genomes of prokaryotic organisms has recently led to the development of new subtyping methods. Multilocus variable number of tandem repeats (VNTR) analysis (MLVA) is based on the number of contiguous DNA repeats present at several loci. Following a repeat-spanning PCR for each locus, the number of repeats can be determined by sequencing or inferred from electrophoresis (molecular weight being correlated with the number of repeats). An MLVA scheme for serotype Typhimurium based on the analysis of five loci (with repeat units of 6 to 33 bp) has been established and evaluated [9]. Unlike PFGE, MLVA is rapid, technically simple and suitable for the processing of large numbers of isolates. It can also distinguish between clonal isolates indistinguishable by PFGE, such as those belonging to the multidrug-resistant DT104 strain. However, this method has several drawbacks. MLVA schemes have been validated (i.e., shown to meet performance and convenience criteria, including the epidemiological concordance required for typing methods for use in bacterial epidemiology [10]) for only two *Salmonella* serotypes, Typhimurium and Enteritidis [11]–[13]. It requires a capillary electrophoresis system and it is difficult to size fragments accurately, as observed in multicenter studies. Finally, these repetitive DNA sequences may evolve too rapidly, leading to changes in repeat numbers during the course of an outbreak [12], [13]. MLVA is therefore often used in addition to existing subtyping methods, such as PFGE or phage typing.

Jansen *et al*. identified a new family of repeated DNA sequences, named CRISPR (clustered regularly interspaced short palindromic repeats) in many prokaryotes [14]. This family is characterized by 24–47 bp DNA direct repeats (DRs), separated by variable 21–72 bp sequences called “spacers” [15], [16]. A “leader sequence” and *cas* (CRISPR-associated sequence) genes are often identified adjacent to the CRISPR locus. Since the middle of the 1990s, the CRISPR locus of *Mycobacterium tuberculosis* has been extensively studied and the high degree of polymorphism of its spacer content has led to the development of a subtyping method known as spoligotyping [17]. Subtyping methods based on analyses of the spacers of CRISPR loci have since been developed for bacteria of medical interest, such as *Yersinia pestis* [18], *Corynebacterium diphtheriae* [19] and *Campylobacter* [20]. CRISPR seem to confer resistance to foreign DNA, such as plasmids and phages, and the newly integrated spacers are derived from the invading DNA [21], [22]. Interestingly, these spacers are integrated into the CRISPR locus in a polarized manner [18], [21]. The spacer content of a strain therefore reflects previous DNA introductions and can provide evolutionary information.

Several studies have reported the presence of two CRISPR loci in *Salmonella* [14], [23], [24]. We previously showed, in a preliminary study of 400 *Salmonella enterica* and *Salmonella bongori* reference strains and isolates from 56 serotypes, that CRISPR polymorphisms (i.e., spacer content) were strongly correlated with serotype and subtype [25]. Two studies recently suggested that CRISPR loci might provide information useful for typing [26], [27]. However, these studies considered only a limited number of serotypes from a single geographic area.

We aimed to demonstrate that CRISPR polymorphism analysis is an efficient and powerful alternative to both serotyping and PFGE methods. We first analyzed the spacer content of the two *Salmonella* CRISPR loci in a large global collection of reference strains and well documented isolates belonging to 130 serotypes of all species and subspecies, focusing particularly on the serotypes most frequently involved in human infections. Analysis of the distribution of the >3,800 unique spacers identified showed that spacer content was strongly correlated with both serotype and multilocus sequence typing (MLST) type. Furthermore, the microevolution of spacer content facilitated the robust discrimination of subtypes within most serotypes, including the most prevalent serotypes, Typhimurium and Enteritidis.

We also present here three applications of CRISPR polymorphisms for *Salmonella* surveillance. In particular, we describe a novel high-throughput subtyping assay for serotype Typhimurium (and its emerging monophasic 1,4,[5],12:i:- variant). This bead-based liquid hybridization assay is both rapid and easy to carry out, and is therefore highly suitable for use in public health laboratories.

## Results

### *In silico* Analysis of the Organization and Structure of CRISPR Loci in *Salmonella*

**Figure 1:**
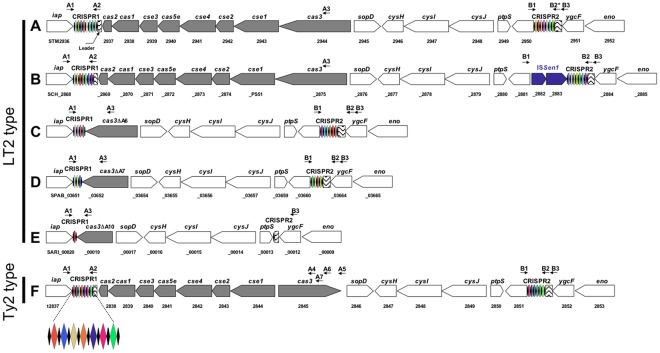
CRISPR/Cas system structures from 39 available genome sequences for *S. enterica* and *S. bongori*. Two CRISPR loci (CRISPR1 and CRISPR2) are present in all genomes. The CRISPR-associated (cas) genes *cas2*, *cas1*, *cse3*, *cas5e*, *cse4*, *cse2*, and *cas3 genes* of the “Ecoli” subtype [28] are located between the CRISPR loci. The most frequent structure, A, is represented by *S. enterica* serotype Typhimurium strain LT2. Structures B to E are represented by *S. enterica* serotypes Choleraesuis strain SC-B67, Javiana strain GA_MM04042433, Paratyphi B strain SPB7, and *S. enterica* subsp. *arizonae* serotype 62:z4,z23:- strain CDC346-86, respectively. Structure F is represented by *S. enterica* serotype Typhi strain Ty2. Black diamonds represent direct repeats, with colored diamonds indicating spacers. The CRISPR1 locus of serotype Typhi strain Ty2 is enlarged. The primers used to amplify and sequence the CRISPR loci for the spacer inventory are indicated by horizontal arrows.

Two CRISPR loci, CRISPR1 and CRISPR2, were separated by less than 20 kb in all 39 complete genomes of *S. enterica* and *S. bongori* analyzed (Figure 1, Table 1). The CRISPR1 locus was located downstream from the *iap* gene, whereas CRISPR2 was located upstream from the *ygcF* gene. The ordered CRISPR-associated (*cas*) genes belonging to the Ecoli subtype defined by Haft *et al.* [28] were located between the CRISPR loci: *cas2*, *cas1*, *cse3*, *cas5e*, *cse4*, *cse2*, and *cas3*. Following the *cas* genes were *sopD* (encoding a secreted effector protein), *cysH* (encoding a phosphoadenosine phosphosulfate reductase), *cysI*, *cysJ* (both encoding sulfite reductase subunits), *ptpS* (encoding pyruvyl tetrahydrobiopterin synthase) and an ORF encoding a putative metal-dependent hydrolase. Structure A was the most frequent, and was observed in 26 (67%) genomes of *S. enterica* subsp. *enterica* (including representative serotype Typhimurium strain LT2), *S. enterica* subsp. *diarizonae* and *S. bongori*. Structure B, which was found only in serotype Choleraesuis SC-B67, differed from structure A by an insertion sequence, IS*Sen1,* immediately upstream from CRISPR2. Structure F, which was found in nine genomes of *S. enterica* subsp. *enterica* (including representative serotype Typhi strain Ty2) differed from structures A and B in having a different orientation of the *cas3* gene and in terms of the degree of similarity of Cas proteins (40 to 85%, depending on the Cas proteins considered; data not shown) [23]. In structures C, D and E (found in *S. enterica* serotype Paratyphi B strain SPB7, *S. enterica* subsp. *arizonae* serotype 62:z4,z23:- strain CDC346-86, and *S. enterica* serotype Javiana strain GA_MM04042433, respectively), there was a deletion beginning at the end of the last DR of CRISPR1 and encompassing all *cas* genes with the exception of a 5′ remnant of *cas3,* which was in the same orientation as that in structure A.

**Table 1:** CRISPR loci detected in the 39 available genomes of *Salmonella*.

Strain	Source (accession no.)	CRISPR structure	CRISPR1 coordinates	CRISPR2 coordinates
***S. enterica*** ** subsp. ** ***enterica*** ** serotype:**				
Agona strain SL483	GenBank (CP001138)	A	2988105-2989231 (18)	3005517-3006033 (8)
Choleraesuis strain SC-B67	GenBank (AE017220)	B	3031533-3031805 (4)	3049243-3049698 (7)
Dublin strain CT_02021853	GenBank (CP001144)	A	3121101-3121251 (2)	3137409-3137742 (4)
Enteritidis strain P125109	GenBank (AM933172)	A	2961370-2961886 (8)	2978038-2978677 (10)
Gallinarum strain 287/91	GenBank (AM933173)	A	2952175-2952325 (2)	2968478-2969117 (10)
Hadar strain RI_05P0661	GenBank (ABFG00000000)	F	NA (28)	NA (29)
Hadar strain “Sanger”1	Sanger Institute2	F	NA (28)	NA (30)
Heidelberg strain SL476	GenBank (CP001120)	A	3051217-3052879 (28)	3069137-3070263 (18)
Heidelberg strain SL4861	GenBank (ABEL00000000)	A	NA (26)	NA (18)
Infantis “Sanger” 1	Sanger Institute2	A	NA (31)	NA (14)
Javiana strain GA_MM040424331	GenBank (ABEH00000000)	C	NA (6)	NA (12)
Kentucky strain CDC1911	GenBank (ABEI00000000)	A	NA (19)	NA (18)
Kentucky strain CVM291881	GenBank (ABAK00000000)	A	NA (18)	NA (17)
Newport strain SL254	GenBank (CP001113)	F	3054859-3056473 (26)	3073142-3074328 (19)
Newport strain SL3171	GenBank (ABEW00000000)	A	NA (12)	NA (18)
Paratyphi A strain ATCC 9150	GenBank (CP000026)	F	2889569-2889902 (5)	2906453-2906664 (3)
Paratyphi A strain AKU_12601	GenBank (FM200053)	F	2885105-2885560 (7)	2902111-2902322 (3)
Paratyphi B strain SPB7	GenBank (CP000886)	D	3041329-3041479 (2)	3050804-3051137 (5)
Paratyphi C strain RKS4594	GenBank (CP000857)	A	3010604-3011242 (10)	3028681-3029258 (9)
Saintpaul strain SARA231	GenBank (ABAM00000000)	A	NA (13)	NA (26)
Saintpaul strain SARA291	GenBank (ABAN00000000)	A	NA (19)	NA (7)
Schwarzengrund strain CVM19633	GenBank (CP001127)	F	2981949-2982709 (12)	2999469-3000534 (17)
Schwarzengrund strain SL4801	GenBank (ABEJ00000000)	F	NA (12)	NA (17)
Tennessee strain CDC07-01911	GenBank (ACBF00000000)	A	NA (41)	NA (21)
Typhi strain CT18	GenBank (AL627276)	F	2926182-2926567 (5)	2943123-2943212 (1)
Typhi strain Ty2	GenBank (AE014613)	F	2912041-2912461 (6)	2929017-2929106 (1)
Typhimurium strain LT2	GenBank (AE006468)	A	3076611-3078147 (23)	3094279-3096260 (32)
Typhimurium strain SL1344	GenBank (FQ312003)	A	3099172-3100159 (15)	3116291-3117723 (22)
Typhimurium strain D23580	GenBank (FN424405)	A	3069598-3071012 (22)	3087144-3088271 (18)
Typhimurium strain 14028S	GenBank (CP001363)	A	3096848-3098323 (23)	3114455-3116070 (25)
Typhimurium strain T000240	GenBank (AP011957)	A	3100041-3101393 (21)	3117525-3119506 (32)
Typhimurium strain DT21	Sanger Institute2	A	NA (21)	NA (26)
Typhimurium strain NCTC 133481	Sanger Institute2	A	NA (10)	NA (26)
Virchow strain SL4911	GenBank (ABFH00000000)	A	NA (55)	NA (16)
Weltevreden strain HI_N05-5371	GenBank (ABFF00000000)	A	NA (40)	NA (26)
4,5,12:i:- strain CVM237011	GenBank (ABAO00000000)	A	NA (23)	NA (26)
***S. enterica*** ** subsp. ** ***arizonae*** ** serotype:**				
62:z4,z23:- strain CDC346-86	GenBank (CP000880)	E	25560-25471 (1)	17801-17773 (0)
***S. enterica*** ** subsp. ** ***diarizonae*** ** serotype:**				
61:l,v:1,5,7 strain CDC01-00051	Washington State University3	A	NA (30)	NA (1)
***S. bongori*** ** serotype:**				
66:z41:- strain 12419	Sanger Institute2	A	2791744 -2792992 (20)	2808974 -2810039 (20)

1 Genomes not finished or annotated.

2 These data were provided by Dougan’s group at the Wellcome Trust Sanger Institute and could be obtained from http://www.sanger.ac.uk/resources/downloads/bacteria/salmonella.html.

3 Available from http://genome.wustl.edu/genomes/.

4 NA, not applicable; the number of spacers per locus is indicated in brackets.

The DRs of both CRISPR loci were conserved. They were 29 bp long and had the consensus sequence 5′-CGGTTTATCCCCGCTGGCGCGGGGAACAC-3′. However, some DR variants carrying single-nucleotide polymorphisms (SNPs) with respect to the consensus sequence were observed (Table S1).

There were 705 unique spacers between the DRs in the two loci from the 39 available genomes (Table S2). Depending on the genome, the number of CRISPR1 spacers varied from 1 to 55 (mean 18.6±standard deviation 13.6) and of the number of CRISPR2 spacers varied from 0 (subsp. *arizonae*) to 32 (15.0±9.8). Spacers were typically 32 bp long (681/705). One was 29 bp long, two were 31 bp long, sixteen were 33 bp long, one was 38 bp long (spacer STM18var2, which contained a VNTR) one was 50 bp long, one was 72 bp long and two were 74 bp long (spacers STM7A/7B and STM7A/7Bvar2 of serotype Typhimurium) (see below). Some spacers were common to different serotypes.

While our study was underway, two CRISPR databases (CRISPRdb and CRISPI) went online [15], [29]. These generalist databases containing >1500 prokaryote genomes incorporate various bioinformatics tools, including one for identifying CRISPR sequences in a selected genome. The application of this tool to *Salmonella* genomes resulted in incorrect results for four to six of the 17 genomes present in both databases. CRISPRfinder did not detect the short CRISPR2 locus of serotype Typhi strains Ty2 and CT18, which have a unique spacer (EntB0var1) between two DRs (DR27 and DR), one of which is degenerate (identity of 20/29 bp). CRISPRfinder detected three CRISPR in serotype Typhimurium strain LT2 and serotype Heidelberg strain SL476. The CRISPR1 locus was actually artificially split into two CRISPR, due to the presence of an unusual fused spacer-DR unit (STM7A/7B, see below). The CRISPI tool detected no CRISPR in four genomes and only one CRISPR in two others. The CRISPR loci identified in all six genomes were short (1 to 6 spacer-DR units) in our study, confirming that the CRISPI tool is not suitable for detecting short CRISPR loci. Thus, although bioinformatics tools are undoubtedly useful for screening for CRISPR within genomes, careful manual inspection is required to complete the analysis for a given species.

### Spacer Content is Strongly Associated with Serotype and MLST

PCR amplification of CRISPR1 with primers A1 and A2 generated a product of between 400 bp and 3 kb in size, in 639 of 744 strains and isolates. By contrast PCR amplification of CRISPR2 with primers B1, B2 and B3 generated a product of between 500 bp and 3 kb in size in all but subsp. *arizonae* strains and isolates (Figure 1, Tables 2 and S2). Various deletions downstream from CRISPR1 or upstream from CRISPR2 were responsible for amplification failure (see below and Table 3). In one reference strain of serotype Mbandaka, PCR was unsuccessful because the CRISPR1 locus was very large (>6 kb) and contained 124 spacer-DR units.

**Table 2:** Primers used for the spacer content inventory.

Primer	Sequence 5′-3′^1^	Coordinates in *Salmonella* genomes^2^	Function^3^
			
A1	GTRGTRCGGATAATGCTGCC	AE006468 (3076537-3076556) AE014613 (2911967-2911986)	Forward primer for amplification of CRISPR1 or for combined amplification of both CRISPR1 and CRISPR2 loci
A2	CGTATTCCGGTAGATBTDGATGG	AE006468 (3078306-3078284) AE014613 (2912608-2912586)	Reverse primer for amplification of CRISPR1 in 640 (86%) of the isolates
A3	CTATTTTGGRCTRCCGACRATG	AE006468 (3085738-3085717)	Reverse primer for amplification of CRISPR1 in 60 (8.1%) isolates of type A structure
A4	GCAATCGGAGCGATTGATGGC	AE014613 (2920120-2920100)	Reverse primer for amplification of CRISPR1 in 29 (3.9%) isolates of type F structure
A5	TCAACACTCTCTTCACCCAG	AE014613 (2921235-2921216)	Reverse primer for amplification of CRISPR1 in 7 (0.9%) isolates of type F structure
A6	TAACCAGCCCTCTTCTGCCTG	AE014613 (2920910-2920892)	Reverse primer for amplification of CRISPR1 in 2 (0.3%) isolates of type F structure
A7	CGCATCATCAACCGTGTTGCG	AE014613 (2920524-2920504)	Reverse primer for amplification of CRISPR1 in 6 (0.8%) isolates of type F structure
B1	GAGCAATACYYTRATCGTTAACGCC	AE006468 (3094155-3094179) AE014613 (2928893-2928917)	Forward primer for amplification of CRISPR2
B2	GTTGCDATAKGTYGRTRGRATGTRG	AE006468 (3096328-3096303) AE014613 (2929174-2929150)	Reverse primer for amplification of CRISPR2 for the isolates belonging to subspecies other than arizonae and diarizonae
B3	CTGGCGGCTGTCTATGCAAAC	AE006468 (3096602-3096582) AE014613 (2929448-2929428)	Reverse primer for single amplification of CRISPR2 for the isolates belonging to all subspecies or reverse primer for combined amplification of both CRISPR1 and CRISPR2 loci

1 Degenerate positions: R = G or A; Y = T or C; M = A or C; K = G or T; D = G or A or T; B = G or T or C.

2 AE006468, serotype Typhimurium LT2 strain; AE014613, serotype Typhi Ty2 strain.

3 The primer pairs used for CRISPR1 amplification for each of the 744 strains are indicated in Table S2.

**Table 3:** Serotypes with deletions of the Cas machinery.

Name		Deleted cas2-cas3 region^1^	*cas3* remnant size in bp^2^	Serotypes with such deletion (no. of isolates)
**Type A CRISPR structure**				
ΔA1	3078148-3084080		2622	Stourbridge (7)
ΔA2	3078148-3084337		2365	Kundunchi (1)
ΔA3	3078148-3084606		2096	Choleraesuis (2)
ΔA4	3078148-3084649		2053	Napoli (1)
ΔA5	3078148-3084656		2046	Mbandaka (2)
ΔA6	3078148-3084763		1939	Javiana (4)
ΔA7	3078148-3084995		1707	Abony (1), Paratyphi B (25)
ΔA8	3078148-3085040		1662	Enteritidis (1)
ΔA9	3078148-3085289		1413	subsp. indica 6,7:z41∶1,7 (1)
ΔA10	3078148-3085385		1317	All subsp. arizonae (5)
ΔA11	3078148-3085559		1143	Enteritidis (1)
ΔA12	3078148-3085681		1021	Worthington (12)
**Type F CRISPR structure**				
ΔF1	2912462-2919593		1406 (1899)	subsp. houtenae 48:g,z51:- (1)
ΔF2	2912462-2919670		1329 (1822)	Portedeslilas (1)
ΔF3	2912462-2919881		1118 (1611)	Newport (5)
ΔF4	2912462-2919890		1109 (1602)	Johannesburg (1), Urbana (2)
ΔF5	2912462-2919901		1098 (1591)	9,12:l,v:- (1), Arechavaleta (1), Brandenburg (3), Chester (1), Glostrup (1), Goettingen (1), Gueuletapee (1), Maracaibo (1), Miami (4), Panama (3), Pomona (2), Reading (1), Rubislaw (1), Sandiego (1)
ΔF6	2912462-2920094		905 (1398)	Albany (1), Duesseldorf (1)
ΔF7	2912462-2920154		845 (1338)	Choleraesuis (1)
ΔF8	2912462-2920810		189 (682)	subsp. houtenae 1,40:z4,z24:- and 44:a:- (2)
ΔF9	2912462-2920937		62 (555)	Bardo (1), Newport (1)
ΔF11	2912462-2921077		0 (415)	Carrau (1), Madelia (1)
ΔF12	2912462-2921188		0 (303)	Newport (3)

1 The coordinates of the deleted regions of isolates with type A CRISPR structure and those of isolates with type F CRISPR structure are based on *S. enterica* serotype Typhimurium strain LT2 (GenBank AE006468) and serotype Typhi strain Ty2 (GenBank AE014613) genomes, respectively. The reverse primers used for these isolates are indicated in Table 2.

2 The *cas3* gene of serotype Typhimurium LT2 strain is 2663 bp in size, whereas that of serotype Typhi strain Ty2 is 2207 bp in size, due to a frameshift leading to a premature stop codon. The sizes of the *cas3* gene remnant are shown in brackets, not taking into account the serotype Typhi-specific frameshift.

More than 3,800 different spacers (mean length of 32 nucleotides) were identified in the 39 available genomes and 744 strains and isolates tested (Tables S2, S3, S4). The number of spacers present in a given strain ranged from 1 to 124 for CRISPR1, and from 0 (subsp. *arizonae*) to 50 for CRISPR2 (Table S3). Two rare groups of strains displayed low levels of correlation between spacer content and serotype or MLST type. First, all the reptile-associated subsp. *arizonae* strains had the same single CRISPR1 spacer, despite the diversity of their serotypes and STs. Second, some reptile-associated subsp. *enterica* serotypes, such as Urbana, Johannesburg, Reading, Pomona, Gueuletapee, Rubislaw, Goettingen and Sandiego had a limited set of spacers shared between these serotypes that belonged to a highly recombinogenic group known as clade B [30] or lineage 3 [31]. Both groups of strains displayed deletions (ΔA10 for subsp. *arizonae* serotypes and ΔF4 and ΔF5 for clade B subsp. *enterica* serotypes) of the *cas* genes (see below). However, with the exception of these two groups, spacer content was strongly correlated with serotype and/or MLST type for 730 of 744 (98.1%) strains. Moreover, for polyphyletic serotypes comprising unrelated MLST groups, spacer content was strongly correlated with the population structure defined by MLST (Table S2). For example, the CRISPR data for three recently described genetic lineages of serotype Newport [32], gave correct serotype recognition and genetic lineage assignment (Tables 4 and S5).

**Table 4:** Comparison of CRISPR1 spacer content with the population structure of *S. enterica* serotype Newport, as assessed by MLST.

Lineage	Strain	MLST^1^	CAS type(deletion)^2^	CRISPR1 spacer content^3^
**Newport-I**	00-4093	ST156	Ty2 (ΔF3)	Ind1var1-H1-H2-H3-H4-H5-H7-H8-H9-N32-H14-N33-N51-N52-N53-Bovis3-H15-N54-N55-DueB1-N56-N60-N61-N57-N58-N59
	01-2174	ST156	Ty2 (ΔF3)	Ind1var1-H1-H2-H3-H4-N55-DueB1-N56-N60-N61-N57-N58-N59
	00-973	ST166	Ty2 (ΔF3)	Ind1var1-H1-H2-H3-H4-H5-H7-H8-H9-N32-H14-N33-N51-N52-N53-Bovis3-H15-N54-N55-DueB1-N56-N62-N60-N61-N57-N58-N59
	04-2487	ST166	Ty2 (ΔF3)	Ind1var1-H1-H2-H3-H4-H5-H7-H8-H9-N32-H14-N33-N51-N52-N53-Bovis3-H15-N54-N55-DueB1-N56-N62-N60-N61-N57-N59
	39/64	ST166	Ty2 (ΔF3)	Ind1var1-H1-H2-H3-H4-H5-H7-H8-H9-N32-H14-N33-N51-N52-N53-Bovis3-H15-N54-N55-DueB1-N56-N60-N61-N57-N58-N59
**Newport-II**	10/66	ST45	Ty2	H7-N1-H8-N2-N3-N4-N5-N31-N6-N7-N8-N9-N10-N22-N12-N14-N15-N16-N17-N18-N21
	00-4165	ST45	Ty2	H7-N1-H8-N2-N3-N4-N5-N6-N7-N8-N9-N10-N22-N23-N24-N11-N12-N13-N14-N15-N16-N17-N18-N19-N20-N21
	02-7891	ST45	Ty2	H7-N1-H8-N2-N3-N4-N5-N6-N7-N8-N9-N10-N22-N23-N24-N11-N12-N13-N14-N15-N16-N17-N18-N19-N20-N21
	04-9597	ST45	Ty2	H7-N1-H8-N2-N3-N4-N5-N6-N7-N8-N9-N10-N22-N23-N24-N11-N12-N13-N14-N15-N16-N17-N18-N19-N20-N21
	SL254	ST45	Ty2	H7-N1-H8-N2-N3-N4-N5-N6-N7-N8-N9-N10-N22-N23-N24-N11-N12-N13-N14-N15-N16-N17-N18-N19-N20-N21
	01-2010	ND	Ty2	H7-N1-H8-N2-N3-N4-N5-N6-N7-N8-N9-N10-N22-N23-N24-N11-N12-N13-N14-N15-N16-N17-N18-N19-N20-N21
	03-3224	ND	Ty2	H7-N1-H8-N2-N3-N4-N5-N6-N7-N8-N9-N10-N22-N23-N24-N11-N12-N13-N14-N15-N16-N17-N18-N19-N20-N21
	10/56	ST46	Ty2	H7-N1-H8-NB25var1-N26-N27-N2-N28-N3-N4-N5-N31-N6-N7-N8-N24-N11-N12-N13-N29-N30-N16-N17-N18-N19-N21
	50K	ST31	Ty2 (ΔF12)	H7-N1-H8-NB25var1-N26-N27-N2-N28-N3-N4-N5-N31-N6-N7-N8-N9-N23-N24-N11-N12-N13-N29-N30-N14-N15-N16-N17-N18-N19-N20
	04-1198	ST31	Ty2 (ΔF12)	H7-N1-N17-N18-N19-N20
	50/3	ST31	Ty2 (ΔF12)	H7-N1-H8-NB25var1-N26-N27-N2-N28-N3-N4-N5-N31-N6-N7-N8-N9-N23-N24-N29-N30-N14-N15-N16-N17-N18-N19-N20
	2/58	ST211	Ty2 (ΔF9)	H7-N1-H8-NB25var1-N26-N27-N2-N28-N3-N19-N20-N21
**Newport-III**	05-0815	ST118	LT2	STM1var1-N45-N34-N35-N36-N37-N38-N39-N40-N46-N47-N48-N49-N42-N43-N44
	4/51	ST118	LT2	STM1var1-N45-N35-N36-N37-N38-N39-N40-N46-N47-N48-N49-N50-N42-N43
	03-8748	ST118	LT2	STM1var1-N45-N34-N35-N36-N46-N47-N48-N42-N43-N44
	SL317	ST5	LT2	STM1var1-N34-N35-N36-N37-N38-N39-N40-N41-N42-N43-N44

1 ND, Not done.

2 The deletions are named according to Table 3.

3 Due to space constraints, the spacer names Newp and Had are abbreviated to N and H, respectively.

**Figure 2:**
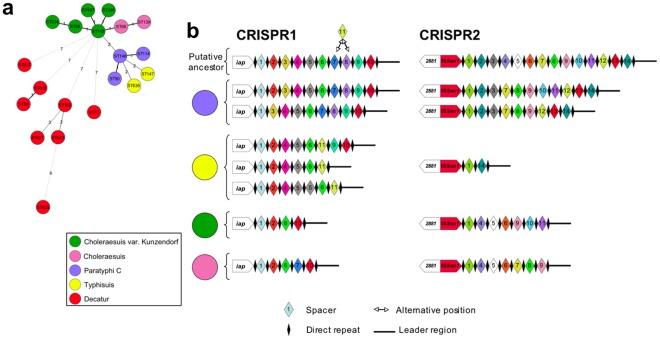
Multilocus sequence typing and CRISPR spacer content of 34 *S. enterica* strains and isolates with the antigenic formula 6,7:c:1,5. Based on additional biochemical characters, we can identify five subserotypes: Choleraesuis *sensu stricto*, Choleraesuis variant Kunzendorf, Paratyphi C (human-restricted), Typhisuis (pig-restricted) and Decatur. MLST (a) and CRISPR data (b) show that Choleraesuis, Paratyphi C and Typhisuis share a common ancestor, whereas Decatur is made up of at least five unrelated populations. The numbers in panel “a” correspond to the allelic difference between STs. The size of the circle is not correlated with the number of strains with the corresponding ST. The exact name of the spacers and the spacer content of Decatur strains from panel b can be found in Table S2.

Most of the spacers were unique to particular serotypes. The degree of spacer sharing varied among groups of serotypes identified as closely related on the basis of MLST. For example, in serotypes such as Typhimurium (4:i:1,2) Heidelberg (4:r:1,2) and Kisangani (4:a:1,2), all the spacers on the *iap* gene side tended to be the same whereas the spacers present on the leader side tended to be more serotype-specific. For isolates of the ST11 group of serotype Enteritidis (9,12:g,m:-) and the closely related Gallinarum (9,12:-:-) and Dublin (9,12:g,p:-) serotypes, only a subset of common spacers was identified (Tables 5 and S6). This was also the case for the complex group of “bioserotypes” Paratyphi C, Choleraesuis (*sensu stricto* and variant Kunzendorf), and Typhisuis, which have the antigenic formula 6,7:c:1,5 in common, indicating descent from a common ancestor, consistent with MLST data (Figure 2). The presence of IS*Sen1* at the same position upstream from CRISPR2 in these bioserotypes is also consistent with the hypothesis of a common ancestor. By contrast, the polyphyletic serotype Decatur (formerly known as serotype Choleraesuis variant Decatur which also has an antigenic formula of 6,7:c:1,5) did not have an IS*Sen1* element upstream from CRISPR2 and included various spacers not found in Paratyphi C, Choleraesuis and Typhisuis (Table S2).

**Table 5:** CRISPR1 spacer content in various O:9 and O:2 serotypes.

Serotype	Antigenic formula	Biotype	MLST	No. of isolates	CRISPR1 spacer content
Enteritidis	9,12:g,m:-		ST11 group1		
				1	Ent1
				2	Ent1-Dub1-Ent3-Ent8
				2	Ent1-Ent2-Ent3-Ent4-Ent4-Ent5-Ent6-Ent7-Ent8
				1	Ent1-Ent2-Ent3-Ent4-Ent5
				76	Ent1-Ent2-Ent3-Ent4-Ent5-Ent6-Ent7-Ent8
				7	Ent1-Ent2-Ent3-Ent4-Ent5-Ent6-Ent7-Ent9-Ent8
				10	Ent1-Ent2-Ent3-Ent4-Ent5-Ent7-Ent8
				7	Ent1-Ent2-Ent3-Ent4-Ent5-Ent7-Ent9-Ent8
				1	Ent1-Ent2-Ent3-Ent4-Ent5var1-Ent6-Ent7-Ent9-Ent8
				92	Ent1-Ent2-Ent3-Ent5-Ent6-Ent7-Ent9-Ent8
				1	Ent1-Ent2-Ent5-Ent6
				1	Ent1-Ent2-Ent5-Ent6-Ent7-Ent8
				1	Ent1-Ent2var1-Ent3-Ent4-Ent5-Ent6-Ent7-Ent8
				51	Ent1-Ent2var1-Ent3-Ent4-Ent5-Ent6-Ent7-Ent9-Ent8
				2	Ent1-Ent3-Ent5-Ent6-Ent7-Ent9-Ent8
			Other STs		
			ST180	1	Ent1-Ent5-Ent6-Ent10-Ent11-Ent7-Ent9-Ent12
			ST180	1	Ent1-Ent5-Ent6-Ent10-Ent11-Ent7var1-Ent12
			ST180	1	Ent1-Ent5-Ent6-Ent10-Ent7-Ent12
			ST6	1	Ent16-Ent17-Ent18-Ent19-Ent20?//?Ent353
			ST77	1	STM1-Ent13-EmeB14-Ent14-CholB19-Ent15
	9,12:g,m,p:-		ST74	1	Ent1-Ent5-Ent6-Ent10-Ent11-Ent7-Ent12
	9,12:g,m,p:-		ST74	1	Ent1-Ent5-Ent6-Ent11-Ent7-Ent12
	9,12:g,m:1,7		ST746	1	Ent36-Mba9-Ent37-Ent38
Blegdam	9,12:g,m,q:-		ST739 (ST11 SLV)	1	Ent1-Ent2-Ent3-Ent5-Ent6-Ent7-Ent9-Ent8
Rosenberg	9,12:g,z85:-		ST11	2	Ent1-Ent2-Ent3-Ent4-Ent5-Ent6-Ent7-Ent8
			ST11	1	Ent1-Ent2-Ent3-Ent4-Ent4-Ent5-Ent6-Ent7-Ent8
Dublin	9,12:g,p:-		ST10	4	Ent1-Dub1
			ST73	2	Ent1-Dub1
Gallinarum	9,12:-:-				
		Gallinarum	ST78	7	Ent5-Ent6var1
		Pullorum	ST92	3	Ent3-Ent4
		Pullorum	ST747 (ST92 SLV)	1	Ent3-Ent4
		Pullorum	ST92	1	Ent1-Ent3-Ent4
		Duisburg4	ST762	2	Ent1-Ent3-Ent4
Nitra	2,12:g,m:-		ST11	2	Ent1-Ent2-Ent3-Ent4-Ent5-Ent6-Ent7-Ent8
			ST11	1	Ent1-Ent2-Ent3-Ent5-Ent6-Ent7-Ent9-Ent8
Kiel	2,12:g,p:-		ST10	3	Ent1-Dub1

1 ST (sequence type) 11 group consists of ST11 and its single-locus variants (SLV).

2 Includes the 5 ST136 “Danysz”» strains used as rodenticides.

3 Ent20−//−Ent35, 15 unique spacers are located between Ent20 and Ent35 (see Table S2).

4 Serotype Gallinarum biovar Duisburg is different from serotype Duisburg.

### Deletions Downstream from CRISPR1 and Upstream from CRISPR2 in some *Salmonella* Populations

For representative strains or isolates for which no PCR was obtained from CRISPR1 or CRISPR2, we carried out a long-range PCR encompassing both CRISPR loci, with primers A1 and B3. Amplicons were obtained from all isolates and were between 10 kb and 20 kb in size. DNA sequencing of the ends of the PCR products showed that amplification failure resulted principally from large deletions affecting the *cas* genes, ending at the *cas3* gene and preventing the annealing of the A2 primer (Table 3). For subsp. *arizonae* and serotypes Paratyphi B and Javiana, these deletions were consistent with data for the three corresponding available genome sequences and from Fricke *et al.* [24]. Such deletions were also observed in other serotypes or populations within a single serotype, but they were of different types. We therefore designed and validated new CRISPR1 reverse primers binding to the residual region of the *cas3* gene from structure A (A3 and A4) or F (A5 to A7) (Table 2). Due to a deletion upstream from CRISPR2, the B1 primer did not bind to DNA from subsp. *arizonae*. However, the available genome sequence and sequencing of the long PCR fragment generated with the A1 and B3 primers from four other isolates showed that there was only one DR (DR68) and no CRISPR2 spacer in this subspecies.

### Microvariations of the Spacer Content Discriminate below the Serotype Level

We observed stable microvariation (duplication, triplication, loss or gain of spacers, presence of SNP variant spacers or VNTR variant spacers) within the strains of monophyletic serotypes. This was the case for the most prevalent serotype worldwide, Typhimurium and its monophasic 1,4,[5],12:i:- variant, for which we analyzed eight genomes and 150 well characterized isolates collected between 1947 and 2010 (Table S7).

*In silico* analysis of genome sequences identified 28 unique spacers within CRISPR1. This number increased to 40 after analysis of the additional 150 isolates (between 6 and 31 spacers per isolate). The order of spacers was strictly conserved. Most were 32 bp (31/40) or 33 bp (2/40) long. Four of the 40 spacers had SNP variants, one (STM18) had four VNTR variants (26 to 50 bp), and the 74 bp STM7A/7B and STM7A/7Bvar2 spacers contained a 28 bp spacer fused to 14 bp from the end of a DR followed by a classical 32 bp spacer (Figure S1). This fusion spacer-DR unit may have been generated accidentally during the process of spacer acquisition.

*In silico* analysis identified 36 unique spacers within CRISPR2. This number increased to 39 upon sequencing of the additional 150 isolates (between 4 and 40 spacers per isolate). All spacers were 32 bp (38/39) or 33 bp (1/39) long. The 39 spacers included only two variant spacers (SNP variants, all the other spacers being unrelated). As for CRISPR1, the variability of CRISPR2 was due to duplication of a single spacer (STMB13)-DR unit and/or to deletion of single or contiguous spacer-DR units.

The order of the spacers was strictly conserved in all but four alleles of CRISPR2 (8 isolates). The variability of CRISPR1 and CRISPR2 spacer content resulted from the duplication of single spacer (STM5, STM8, STM22, STM28, and STMB13)-DR units and/or the deletion of single or contiguous spacer-DR units. This microvariation resulted in 57 CRISPR1 and 62 CRISPR2 alleles or into 83 CRISPR1-CRISPR2 combined alleles, thus providing a higher resolution than other subtyping methods, such as PFGE. Particular populations, such as multidrug-resistant (MDR) DT104 isolates [33], African MDR ST313 isolates [34], and ciprofloxacin-resistant isolates [35], each had typical CRISPR alleles.

*In vitro* stability experiments showed no difference in spacer content for either of the CRISPR loci between the five original serotype Enteritidis isolates and their derived cultures after one month or two months with daily passages. Furthermore, the genome sequences of widely used laboratory strains of serotype Typhimurium, LT2 (isolated in 1947) and SL1344 (isolated in the 1970s), showed these strains to have the same spacer content (15 to 32 per locus) as strains LT2 and SL1344 available in our laboratory. The stability of this marker was also assessed by performing the microbead-based CRISPOL assay (see below) one year later on fresh cultures (grown from single colonies) of the 150 serotype Typhimurium and monophasic 1,4,[5],12:i:- isolates (mean of 41±9 spacers per isolate). One isolate had a discordant CT with respect to the initial spacer content determined by sequencing. We resequenced both CRISPR loci in this subcultured isolate and found that a single CRISPR1 spacer-DR unit had been lost. During the systematic CRISPOL testing of all serotype Typhimurium and monophasic isolates obtained between January 1 2010 and July 7 2010, 43 duplicate and four triplicate isolates obtained from the same patients on different days were analyzed (see below). All but one had concordant CTs. The final isolate had lost a CRISPR1 spacer-DR unit that was present in the other two isolates from the same patient. Thus, spacer content is, at least in serotypes Enteritidis and Typhimurium, stable enough for use in surveillance and outbreak investigation. However, although rare and often minor (single spacer variant of the original CT in both cases detected), CRISPR variation may occur and, whatever its origin, occurring before or during carriage in the patient or subculture in the laboratory, should be taken into account when defining outbreak-related CTs.

We assessed the discriminatory power of the method, an important parameter for surveillance purposes, by comparing CRISPR spacer diversity with classical first-line (PFGE, phage typing) and second-line (MLVA) subtyping methods for a subset of 50 randomly selected clinical isolates collected in 2002 [33]. We found 17 different alleles for CRISPR1 (Simpson’s discrimination index (DI) = 0.84), 23 for CRISPR2 (DI = 0.84), and 26 if a combination of the two loci was considered (DI = 0.88). These isolates gave 26 *Xba*I-PFGE profiles (DI = 0.87) and 14 phage types (DI = 0.74). For prevalent MDR DT104 isolates, the discriminatory power was higher for combined CRISPR analysis (5 profiles, DI = 0.64) than for PFGE (also 5 profiles, but DI = 0.38). The best discrimination was that achieved with the five-locus loci MLVA method, for which all the DT104 isolates had different profiles (DI = 1). However, it was not possible to amplify some MLVA loci from some isolates (null alleles were seen for STTR3 in 4 isolates, STTR5 in 1 isolate, STTR6 in 3 isolates and STTR10 in 3 isolates) and variations in the number of repeats of some loci were observed in outbreak-related isolates, indicating lower levels of epidemiological concordance, possibly due to the very rapid evolution of these markers or to outbreaks being caused by more than two MLVA types (Table S7).

The spacer content of serotypes Typhimurium and Enteritidis isolates from 10 documented outbreaks was studied by sequencing (Tables S7 and S8) or with the microbead-based CRISPOL assay (see below). In all cases, the outbreak isolates had the same CRISPR type. Epidemiological concordance was thus complete.

Four strains (SARA8, 81-784, 02-7015 and 07-1777) with a known spacer content covering all the spacers identified were tested in every microbead-based CRISPOL experiment. Their CRISPR types were identical in all cases.

In addition to 100% typeability, the other performance criteria [10] for CRISPR analysis, such as stability, discriminatory power, epidemiological concordance and reproducibility, indicated that this was a very powerful method for use in the molecular epidemiology of *Salmonella*.

### Applications of CRISPR Polymorphisms

There are at least three applications of CRISPR polymorphisms of potential interest in clinical microbiology or public health laboratories.

#### Application 1: CRISPR sizing by PCR for the rapid comparison of Salmonella spp isolates

**Figure 3:**
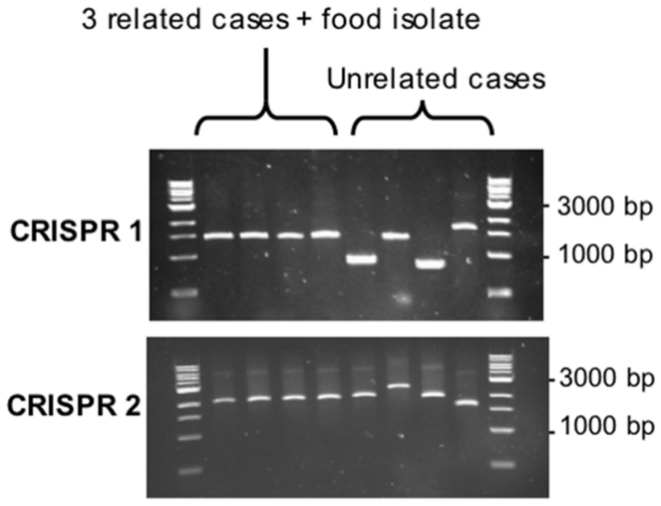
CRISPR sizing by PCR for the rapid comparison of *Salmonella* spp isolates. Results of PCR amplification for 8 *S. enterica* serotype Typhimurium isolates collected from the same city during a single week (cluster E in Table S7). Three cases were from the same food poisoning cluster (the food isolate was also tested), whereas the other cases were unrelated.

The first application is a double-locus PCR assay for the rapid comparison of *Salmonella* isolates. We demonstrate above that variation in the number and type of spacers can be used to track strains, given the discrimination between the most prevalent *Salmonella* serotypes. Remarkably, simple PCR amplification of CRISPR1 and CRISPR2 loci, followed by agarose gel electrophoresis and sizing of the PCR products, differentiated outbreak isolates from non outbreak isolates and was therefore found to be a useful screening approach. For example, for the eight isolates of serotype Typhimurium isolated from the same city during a single week in 2005 (cluster E in Table S7), it was possible to discriminate between four isolates from the same food poisoning cluster and four other isolates unrelated to this cluster (Figure 3). This size variation results from variation in the number of spacer-DR units (total 60 bp), which thus provides some discrimination even in the absence of qualitative information (i.e., the spacer type). Another advantage of this approach is that it does not require prior serotype identification, as sequences from isolates of all serotypes can be amplified by at least one of the two primer pairs used for CRISPR amplification. A different amplicon size for one or both loci demonstrates that the analyzed isolates are unrelated, but it should be borne in mind that a similar amplicon size for both loci does not necessarily imply that the two isolates belong to the same strain. Two unrelated isolates could have the same number of spacers but of different types, and low-level variation in the number of spacers might not be detected on agarose electrophoresis of large PCR products. However, this simple screening approach is suitable for low-capacity public health or hospital laboratories, including those in developing countries, which need to be able to compare several *Salmonella* isolates rapidly in a single experiment and that cannot afford complete serotyping or subtyping by PFGE.

#### Application 2: High-throughput method for subtyping serotype Typhimurium or its monophasic variant in real time: the CRISPOL assay

**Figure 4:**
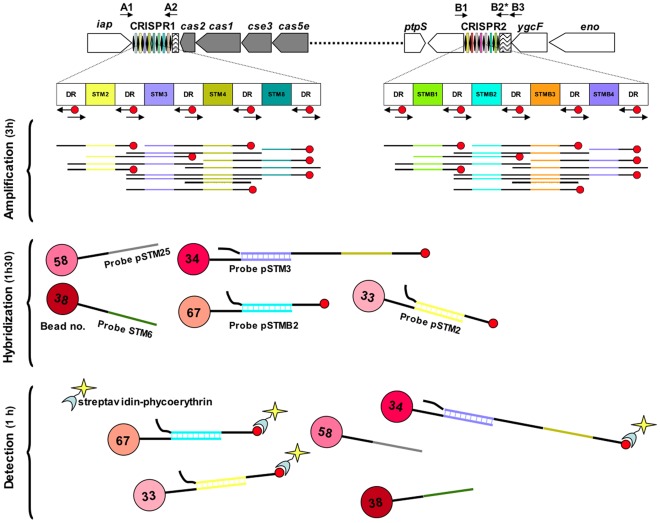
Overview of the bead-based CRISPOL assay for *S. enterica* serotype Typhimurium developed here. The estimated time for each step is based on the testing of 65 isolates in a 96-well plate. The amplification step spans from the preparation of thermolysates to the gel electrophoresis of PCR products.

We present below a second application resulting from the development of a high-throughput method for the real-time subtyping of serotype Typhimurium and its monophasic variant. Based on the 83 CRISPR1-CRISPR2 combined alleles identified above, we developed a bead-based liquid hybridization assay (Luminex® technology), CRISPOL (for CRISPR polymorphism; Figure 4). A 25 to 32 bp capture probe was designed for each of 72 of the 79 spacers identified (Table 6; it was not possible to distinguish between some of the remaining seven spacers by this approach. For example, STMB8var1 has a single SNP located in position 1 of the spacer). Each capture probe was coupled to a defined xMAP bead. We used thermolysates as the DNA template and a single primer pair (including a biotinylated primer) hybridizing to DR sequences to amplify the spacer content of the two CRISPR loci rapidly. The PCR mixture was hybridized with the 72 probe-coupled beads and incubated with streptavidin-phycoerythrin for detection. The Luminex® platform was then used to measure the fluorescence associated with each bead (corresponding to a unique probe/spacer). This method gave a highly robust readout, with mean fluorescence signals of 709 to 5,707 in the presence of the spacer, and of 52 to 193 in the absence of the spacer (Table 7). The positive/negative ratios were between 13 (for a bead for which coupling was not optimal) and 92 (mean of 50.8). It was also easy to identify the four SNP-variant spacers (Table 8). One probe, pSTMB26 had a trimodal distribution, due to an intermediate population (MFI between 300 and 1,200), whereas the positive population had an MFI of more than 3,500. This intermediate population (consisting mostly of emerging European monophasic isolates) contained spacer STMB34, which is partly complementary to pSTMB26. We designed a new probe targeting the other side of spacer STMB26, but this probe was also partly complementary to another spacer, STM28. We resolved this problem by subtracting the value for the control strain 02-7015 (STMB34 positive) from that for probe pSTMB26 in each experiment.

**Table 6:** Serotype Typhimurium spacers and corresponding probes for the CRISPOL assay.

CRISPR locus	Spacer name (position) *	Spacer DNA sequence (5′–3′)‡	Probe name (position) **
1	STM01 (1)	TTTTCAGCCCTTGTCGACTGCGGAACGC CCCT	pSTM01 (1)
1	STM02 (2)	GCGAAATAGTGGGGAAAAACCCCTGGTTAACC	pSTM02 (2)
1	STM03 (3)	TAGGCCTTGATACCATCGCTCGCACC TCGTCA	pSTM03 (3)
1	STM03var1 (3)	TAGGCCTTGATACCATC**A**CTCGCACC TCGTCA	pSTM03var1 (69)
1	STM03var2 (3)	**C** AGGCCTTGATACCATCGCTCGCACCTCGTCA	
1	STM25 (4)	GTTTATTACTGCTTAGTTAATTAATGGGTTGC	pSTM25 (4)
1	STM26 (5)	AGGCGAATAATCTCTAATAGTCTCAATTCGTT	pSTM26 (5)
1	STM27 (6)	TAAATCTGGCGTCGAGACATTCGAAATAGTGC	pSTM27 (6)
1	STM04 (7)	TCTTTTGATTTTGCTGCGATGTTATAACCAGA	pSTM04 (7)
1	STM05 (8)	TATCCACATATACCCGCAATCATATTCAAGAA	pSTM05 (8)
1	STM06 (9)	AATCACTGCGGGGGTATTTAGCGGAAACGGCT	pSTM06 (9)
1	STM07A (10)	GATCGAGTAACGTGCGCTGGAACGCGTCGGCGCGGGGAACAC	
1	STM07B (10)	AATTAAAGCCGAGGGTGGCACCGCGC CTTATT	pSTM07 (10)
1	STM07Bvar2 (10)	AATTAAAGCCGAGGGTGG**T**ACCGCGC CTTATT	pSTM07var2 (70)
1	STM08 (11)	GCACCTCGAAACGGTTTTAAAACAC TACCGTTT	pSTM08 (11)
1	STM09 (12)	TGGACCGATGGGGCCAACATCGCCGAACGTGG	pSTM09 (12)
1	STM10 (13)	GTTACGTTCGGTAAATGGAAAGCGGCGAATAT	pSTM10 (13)
1	STM11 (14)	CCAGAAAGTGCCGGTAGTGCCTGATGAACGAC	pSTM11 (14)
1	STM12 (15)	CGCGCCCACTTCCGTAAAATACAGA TAATCCA	pSTM12 (15)
1	STM12var1 (15)	CGCGCCCACTTCCGTAA**G**ATACAGAT AATCCA	pSTM12var1 (71)
1	STM28 (16)	GGCAGCGGGCGAGGCAAACACATTC GGGGCGT	pSTM28 (16)
1	STM13 (17)	GGTAATTTCTCATCTAACAGCCTGTACGCCTC	pSTM13 (17)
1	STM14 (18)	GAATCTAATGCAACAGATGAATAAACACG TAA	pSTM14 (18)
1	STM15 (19)	TCTTTATCGTCAATGCGAAATTTTCCGCGACG	pSTM15 (19)
1	STM16 (20)	TCCCATTCACCAACAACAATATCGCCCTGCAA	pSTM16 (20)
1	STM17 (21)	CGTTGCGTCAGGTTGATCCAGTGCGTCAGCGG	pSTM17 (21)
1	STM18 (22)	TCTCGGTCTCGGTCTCGGTCTCGGTAGTGACG	pSTM18 (22)
1	STM18var1 (22)	TCTCGGTCTCGGTCTCGGTCTCGGTCTCGGTCTCGGTCTCGGTAGTGACG	
1	STM18var2 (22)	TCTCGGTCTCGGTCTCGGTCTCGGTCTCGGTAGTGACG	
1	STM18var3 (22)	TCTCGGTCTCGGTCTCGGTAGTGACG	
1	STM18var5 (22)	TCTCGGTCTCGGTCTCGGTCTCGGTCTCGGTCTCGGTAGTGACG	
1	STM19 (23)	ACTTCCTTCAGTCTTAACGATAATCCGCAACG	pSTM19 (23)
1	STM20 (24)	GCAAAATAGCGATGAGCTGGCTACGCCCACTGG	pSTM20 (24)
1	STM29 (25)	AGCCGGCGCGAGCCTGGAGGGTTGAATAATGG	pSTM29 (25)
1	STM30 (26)	CAATCTCGCATTCGTTACCCCACCTGCATTTT	pSTM30 (26)
1	STM21 (27)	GAGGGGATAGGAGTTACGATCCAGCCTGGTTG	pSTM21 (27)
1	STM22 (28)	GTGGTTGCAGACCAATCAGCCCGCCAGCGGTT	pSTM22 (28)
1	STM24 (29)	CAGCACGAAAAATTATTTACTGTCGTTGCTCA	pSTM24 (29)
1	STM31 (30)	TGTAACAGTCCGTCGTTAATCAGCGCGGTGGG	pSTM31 (30)
1	BraB14 (31)	GAAGGTACGGGGAAAACAAAGATTACTCGTTC	pBraB14 (31)
2	STMB0 (1)	ATCTTCATATTGCGTGACGCTGCCGATGAACG	pSTMB0 (32)
2	STMB32 (2)	TCTTTATCAGCTAACCATTTCCAGAACTCGTC	pSTMB32 (33)
2	STMB01 (3)	TATAATATGAATTAATTTTTGCGCATAACCTG	pSTMB01 (34)
2	STMB01var1 (3)	TATAATATGAATTAATTTTTGCG**T**ATAACCTG	
2	STMB02 (4)	TGCCCGTTCTGCCTCTTCGCACTCTCGATCAA	pSTMB02 (35)
2	STMB03 (5)	TGCGTAATGGGCTACCTGAACTTCACATATCC	pSTMB03 (36)
2	STMB04 (6)	ATTAAGCGCGCAAAGTTTGGGTTAATTGGACA	pSTMB04 (37)
2	STMB05 (7)	CGTATTCGTCACACAGCCCCGTCCAGAAATGA	pSTMB05 (38)
2	STMB06 (8)	TAACGAACTGAATAAAATGTCAGAAAGTGACG	pSTMB06 (39)
2	STMB07 (9)	GCAGCTTAGCGACGAAATTAAAACCGAACTCAC	pSTMB07 (40)
2	STMB08 (10)	TGCCAGTGACTACAGAAGCGTCGCTATCGGGG	pSTMB08 (41)
2	STMB08var1 (10)	TGCCAGTGACTACAGAAGCGTC**T**CTATCGGGG	pSTMB08var1 (72)
2	STMB09 (11)	ACCGATAAACAACCGCATAGCCTCTT TCGTTT	pSTMB09 (42)
2	STMB10 (12)	TGCTCAATAACGTCGTAAATAGCGTAAGCTGG	pSTMB10 (43)
2	STMB11 (13)	TATTTCGCCTTCGGCACTGACGTCACCGTCAA	pSTMB11 (44)
2	STMB12 (14)	GTCGCGTTCGTTGCCGGTATAGACCAGCGTCA	pSTMB12 (45)
2	STMB13 (15)	ATCGAATCGAAACCCCAGCCACAGA AATAATT	pSTMB13 (46)
2	STMB14 (16)	GCTCATGTCAAACGCCATCAGCGTTCCGGCAT	pSTMB14 (47)
2	STMB15 (17)	AATCGCCAGCCTCGGAAATATTCCATCCTCCG	pSTMB15 (48)
2	STMB16 (18)	AGGAACTAAACAGCCTGACCGTTGAGGATCTG	pSTMB16 (49)
2	STMB17 (19)	ACCGGACAAATCTTTTTTTTCCTGTTCCTGTT	pSTMB17 (50)
2	HadB20 (20)	GGGCGGTCCCCGGCCTCAATACCGCGCTGACG	pHadB20 (51)
2	STMB34 (21)	TTGAGGTGCCGCTTGCCGTTCTTCT GTTTTTT	pSTMB34 (52)
2	STMB18 (22)	GGGCACTATGAACGGATCGGCGCTGATGCCGG	pSTMB18 (53)
2	STMB19 (23)	GGTAAAGCCACACCATTTTTTATTGACCTCGC	pSTMB19 (54)
2	STMB33 (24)	CTAGGAGGCGTAATGAATACTACGTA TCAAAA	pSTMB33 (55)
2	STMB20 (25)	GTGGTGGCCTCAAATAAATTCGAGCGCTGGAG	pSTMB20 (56)
2	STMB21 (26)	TCGACGTGGACGAGGAGTTACTCAACCGCTGC	pSTMB21 (57)
2	STMB22 (27)	AGCGCCACATGGCCCACCGGCACCACCCGATC	pSTMB22 (58)
2	STMB23 (28)	AAATGACCAAATCAGAAATCTTCACCAAAGCC	pSTMB23 (59)
2	STMB24 (29)	TAATGGCCACAGTAAGTCAAACGGTTCTGGAA	pSTMB24 (60)
2	STMB25 (30)	GAGTCCGGGGGTTATATAGTTATTTA ATGAGC	pSTMB25 (61)
2	STMB26 (31)	TTGGGCGTCGGTTTTTTCAGGTTTAGGTCCGG	pSTMB26 (62)
2	STMB27 (32)	TCAACTGTCAGTTCGTCGTTAGCCAGTAATTC	pSTMB27 (63)
2	STMB28 (33)	CTGAAAACGCATGGAATCCGGTATAAACAGTC	pSTMB28 (64)
2	STMB29 (34)	GATGTAACTGATAGCGAAATATATTGGG ATAA	pSTMB29 (65)
2	STMB30 (35)	GAAACGTAAACAGGGTAAGATACAACTCTGCA	pSTMB30 (66)
2	STMB31 (36)	TGTAAAGGGTGGTCTGGAAGGGGAT CGGCAAA	pSTMB31 (67)
2	STMB35 (37)	TCGTGTGAGGTCGCTGAGAAAAACGGGGCGTA	pSTMB35 (68)

* position of the spacer within the CRISPR1 or CRISPR2 locus.

‡ single polymorphic nucleotides that define spacer variants are shown in bold typeface; probe sequences are underlined.

** position of the probe in the CRISPOL assay.

**Table 7:** Probe responses in the CRISPOL assay (data from 25 sequenced isolates).

Probe name (bead no.)	Spacer absentMedian (MFI)±SD^1^	Spacer presentMedian (MFI)±SD	Ratio^2^ positive/negative
pBraB14 (29)	65±10	3866±570	59
pSTMB35 (30)	70±14	3540±445	51
pHadB20 (31)	62±8	1544±200	25
pSTM01 (32)	53±9	2547±332	48
pSTM02 (33)	81±12	2586±308	32
pSTM03 (34)	56±4	3808±5394	68
pSTM03var1 (35)	52±3	2409±5324	48
pSTM04 (36)	57±8	4187±390	73
pSTM05 (37)	173±13	5216±605	30
pSTM06 (38)	64±4	3019±337	47
pSTM07 (39)	66±11	4580±4944	69
pSTM07var2 (40)	65±13	3283±4714	54
pSTM08 (41)	55±7	709±154	13
pSTM09 (42)	68±11	4065±397	60
pSTM10 (43)	94±17	4036±402	43
pSTM11 (44)	62±9	4564±518	74
pSTM12 (45)	69±11	3343±5154	46
pSTM12var1 (46)	61±8	1173±2804	20
pSTM13 (47)	58±7	3812±539	66
pSTM14 (48)	59±8	4183±381	71
pSTM15 (49)	72±14	3458±452	48
pSTM16 (50)	71±13	4990±1061	70
pSTM17 (51)	89±15	2092±333	24
pSTM18 (52)	79±14	3863±610	49
pSTM19 (53)	68±15	3359±289	49
pSTM20 (54)	65±9	3113±375	48
pSTM21 (55)	74±14	4100±573	56
pSTM22 (56)	71±9	2158±304	30
pSTM24 (57)	50±6	4603±440	92
pSTM25 (58)	60±11	4178±382	70
pSTM26 (59)	62±7	4772±521	78
pSTM27 (60)	59±10	4061±364	69
pSTM28 (61)	61±9	4186±328	69
pSTM29 (62)	61±9	3508±418	58
pSTM30 (63)	73±11	4532±670	62
pSTM31 (64)	68±10	3209±409	47
pSTMB0 (65)	55±12	3952±398	73
pSTMB01 (66)	64±16	5409±674	85
pSTMB02 (67)	65±11	3987±500	62
pSTMB03 (68)	58±9	2508±713	43
pSTMB04 (69)	68±13	4913±558	72
pSTMB05 (70)	60±8	5196±574	87
pSTMB06 (71)	61±8	1802±343	30
pSTMB07 (72)	62±10	4447±477	71
pSTMB08 (73)	69±10	1210±4535	53
pSTMB08var1 (74)	69±10	1266±2515	27
pSTMB09 (75)	66±9	1266±251	19
pSTMB10 (76)	64±7	2629±480	41
pSTMB11 (77)	59±9	2036±279	35
pSTMB12 (78)	73±11	2147±276	30
pSTMB13 (79)	69±14	5142±805	75
pSTMB14 (80)	64±8	2034±289	32
pSTMB15 (81)	67±10	3524±466	53
pSTMB16 (82)	78±12	5062±547	65
pSTMB17 (83)	94±16	2947±437	32
pSTMB18 (84)	80±11	3745±532	47
pSTMB19 (85)	101±13	4570±733	45
pSTMB20 (86)	75±14	4573±483	61
pSTMB21 (87)	73±12	3556±319	49
pSTMB22 (88)	84±16	5707±636	68
pSTMB23 (89)	69±9	5434±642	78
pSTMB24 (90)	71±8	3525±450	50
pSTMB25 (91)	72±10	2039±395	28
pSTMB26 (92)	193±2443	3599±664	19
pSTMB27 (93)	101±15	3464±505	34
pSTMB28 (94)	87±12	3218±391	37
pSTMB29 (95)	87±13	3542±519	41
pSTMB30 (96)	79±10	3805±442	48
pSTMB31 (97)	83±15	2195±304	27
pSTMB32 (98)	83±12	4832±551	58
pSTMB33 (99)	70±8	2143±249	30
pSTMB34 (100)	78±11	3109±513	40

1 MFI, median fluorescence intensity (raw data); SD, standard deviation.

2 The ratio is the positive average median fluorescence intensity (MFI) divided by the negative average MFI.

3 Due to partial identity with spacer STMB34, the signal of pSTMB26 is stronger in isolates containing spacer STMB34 (such as the emergent monophasic population). This has been taken into account by subtracting the MFI of control strain **#**02-7015 from that of pSTMB26 in each experiment. The corrected median is 183±46 with a ratio of 20.

4 Median calculated for 24 isolates.

5 Median calculated for 20 isolates.

**Table 8:** Probe responses in the CRISPOL assay for SNP variants (individual isolates).

Probe name (bead no.)	Isolate #81-299 (STM03 variant 1) Median (MFI)±SD	Isolate #DK4 (STM07 variant 2) Median (MFI)±SD	Isolate #02-277 (STM12 variant 1) Median (MFI)±SD	Isolates #02-3369, #02-7105, #01-1639, #81-482, #81-831 (STMB08 variant 1) Median (MFI)±SD
pSTM03 (34)	3807±89	NA	NA	NA
pSTM03var1 (35)	4573±132	NA	NA	NA
pSTM07 (39)	NA	1944±41	NA	NA
pSTM07var2 (40)	NA	5143±140	NA	NA
pSTM12 (45)	NA	NA	1493±61	NA
pSTM12var1 (46)	NA	NA	4146±85	NA
pSTMB08 (73)	NA	NA	NA	2204±328
pSTMB08var1 (74)	NA	NA	NA	4277±420

Results in triplicate; MFI, median fluorescence intensity (raw data); SD, standard deviation; NA: not applicable.

The repeatability of the CRISPOL assay was assessed by running 30 isolates in triplicate and was high (data for five strains provided in Table S9), with low standard deviations. The assay was also highly reproducible, based on the results for the four control strains (which, together, contained all the known spacers) analyzed in each experiment. A cutoff of five times the value for the background sample (consisting of all reaction components except template DNA, which was replaced with water), which had an MFI of approximately 300, was used to determine whether the result was positive or negative for a given spacer. The distribution of crude MFI values in a typical experiment with 65 isolates showed the clear-cut distinction between negative and positive results for spacers (Figure S2).

The concordance of the CRISPOL assay and sequencing results was 100% for the spacer content of the 150 sequenced isolates. Despite the presence of some genetic variation, such as duplications of spacers, VNTR variation of STM18 and the appearance of SNP variants, which might result in spacers being missed by the CRISPOL assay, no such effect was observed in practice as we found no isolates with identical CTs but with alleles with different sequences.

This method has two major advantages: its rapidity and low cost. It requires 5.5 hours in total, with 2.75 hours of hands-on time, to test 65 isolates from bacterial colonies, at an estimated cost of €4 per sample for reagents and consumables.

**Figure 5:**
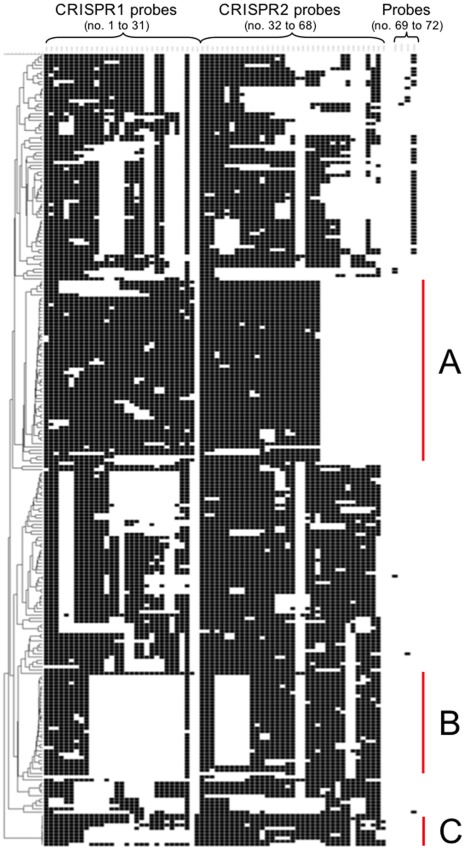
Dendrogram presentation of the 245 distinct CRISPOL types detected among 2,200 isolates of *S. enterica* serotype Typhimurium or its monophasic variant of antigenic formula 1,4,[**5**],12:i:-. Black squares indicate presence of the spacer, as detected by the corresponding probe, whereas white indicates an absence of the spacer. For the determination of CRISPOL types (CTs), each of the 68 spacers was treated as a numerical character indicating absence (0) or presence (1 for all spacers except BraB14, for which an arbitrary value of 10 was assigned) in BioNumerics 6.5 software (Applied Maths, Sint-Martens-Latem, Belgium). Similarities between CTs were assessed by calculating the Pearson product-moment, and a dendrogram was constructed by the unweighted pair group method with arithmetic mean (UPGMA). The four SNP-variant spacers targeted by probes 69 to 72 are shown but were excluded from the phylogenetic analysis, as they were not independent. A indicates a group of profiles derived from CT1, the main type of emerging monophasic isolates. B indicates a group of profiles derived from CT21, which is associated with multidrug-resistant DT104 serotype Typhimurium isolates. C indicates a group of serotype Typhimurium isolates of ST36 that may have one or two specific spacers on the leader side of CRISPR1 (BraB14) and CRISPR2 (STMB35).

**Figure 6:**
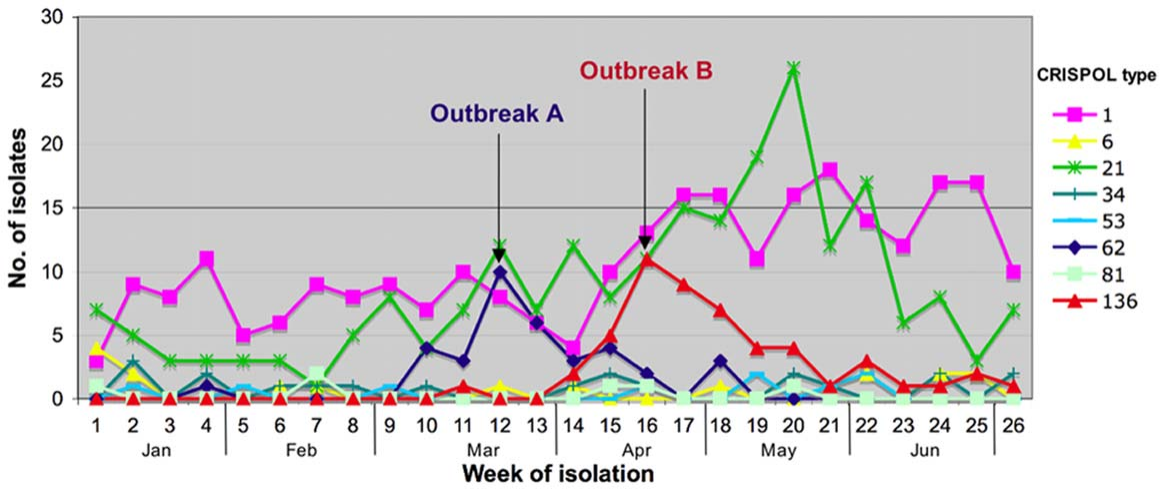
Distribution of selected CRISPOL types of *S. enterica* serotype Typhimurium and *S. enterica* with antigenic formula 1,4,[**5**],12:i :- isolated from humans in France between January 1 and July 4 2010. Over this period, all 1,084 isolates (one per patient) were CRISPOL-typed and two outbreaks were investigated. Outbreak A (≈40 cases) was due to the consumption of a raw milk cheese contaminated with a CT62 highly multidrug-resistant *S. enterica* serotype Typhimurium strain, whereas outbreak B (≈50 cases) was caused by the consumption of a dried pork sausage contaminated with a CT136 *S. enterica* 4,12:i:- strain. The third peak, corresponding to CT21, in May was neither detected nor investigated, as CRISPOL typing was carried out retrospectively.

The application of this method to almost 2,000 serotype Typhimurium and monophasic variant isolates led to the identification of 245 different CRISPOL types (CTs; Figure 5). CT21 and its variants were strongly associated with the MDR DT104 serotype Typhimurium clone (MLST type ST19). Similarly, CT1 and its variants were strongly associated with the emerging European monophasic 1,4,[5],12:i:- variant (MLST type ST34). In total, 1,084 isolates (one per patient) were received at the French National Reference Center for *Salmonella* (FNRC-Salm) between January 1and July 4 2010; 89 CTs were observed among the serotype Typhimurium isolates (n = 677). The two most prevalent types were CT21 (33% of isolates) and CT30 (11.5%); both were associated with the DT104 clone. For monophasic isolates (n = 407), we identified 39 CTs, of which CT1 (50%) and CT9 (14.7%) were the most prevalent. During this period a steady increase in the prevalence of CT1 and three peaks of CT21, CT136 and CT62 (two of which corresponded to documented outbreaks) [36] were observed (Figure 6).

#### Application 3: Development of PCR assays targeting specific serotypes or particular strains

The presence of unique, constant spacers in certain serotypes, such as Typhi and Paratyphi A, should make it possible to develop PCR assays specific for these serotypes. As a proof-of-principle, we have successfully developed and validated such PCRs for serotypes Typhi and Paratyphi A (manuscript in preparation). Moreover, it would be possible to develop a PCR assay for the detection of any strain of interest with a particular spacer content provided that a culture of this strain was available. For strains with no specific spacers (common spacers only), we can use other stable characteristics of the strain, such as the absence of a spacer-DR unit between STM06 and STM24 (e.g., a MDR serotype Typhimurium DT104 strain), to design primers yielding a PCR product of known size.

## Discussion

We demonstrate here that the assessment of CRISPR spacer content is a robust, highly discriminatory and practical method for typing *Salmonella* isolates. Serotyping has been the reference method for *Salmonella* typing for almost 80 years. However, this technique has a number of drawbacks, including low throughput, high costs due to the need for highly trained staff and expensive antisera, and accreditation problems. It is also of limited value for strain discrimination, given the overdominance of a small number of serotypes. There is therefore a clear need for improved methods [37].

Recently developed “molecular serotyping” methods have been proposed as an alternative. These methods mimic serotyping in that they target the genes involved in biosynthesis of the flagellar (*fliC* and *fljB*) [38] and/or the O-polysaccharide (encoded by the *rfb* locus) antigens [2]. However, due to the complexity of the *rfb* locus (8 to 23 kb, including more than 10 open reading frames), it is currently possible to identify only a minority of the 46 O serogroups of *Salmonella* by PCR. These approaches are also subject to all the limitations inherent to serotyping in terms of a lack of discrimination and a lack of polyphyletic group recognition.

MLST is a promising method for defining evolutionarily and epidemiologically meaningful groups of *Salmonella*. A publicly accessible database (http://mlst.ucc.ie/mlst/dbs/Senterica) includes data for more than 4,250 isolates from more than 500 serotypes [39]. Analyses of these data have revealed that most serotypes are probably polyphyletic and therefore do not correspond to natural groups descended from a single ancestor and sharing important host association or virulence features. This recent study highlights the importance of using phylogenetically informative methods recognizing natural groups rather than serotypes. However, MLST has a low discriminatory power and is not suitable for the detection or investigation of outbreaks due to highly prevalent monophyletic serotypes.

The data presented here for a global collection of 783 reference strains and isolates from 130 serotypes of *Salmonella*, including the most common serotypes involved in human infections, show a high degree of CRISPR polymorphism. This polymorphism makes it possible to distinguish between most serotypes and between MLST groups within polyphyletic serotypes. Furthermore, microvariations, such as the loss, acquisition, duplication of spacers or point mutations within spacers, have a strain discrimination capacity similar to that of current gold standard methods, such as PFGE. The CRISPR method can therefore be used for simultaneous typing – defined as the determination of serotype or MLST group – and subtyping. It therefore represents a single alternative to several widely used reference methods: serotyping, PFGE and phage typing. This genetic marker is based on polymorphic DNA sequences of limited length, 0.5 to 3 kb. It therefore has major advantages, in terms of analysis, throughput, standardization, interpretation and data exchange, over current typing and subtyping methods. We believe that this novel approach will constitute a real improvement in the monitoring of *Salmonella* infections, by making it possible to obtain results more rapidly, thereby optimizing surveillance and outbreak detection.

We propose several different strategies for CRISPR genotyping in *Salmonella*. First, determination of the sizes of the two CRISPR loci by PCR can be used as an initial screen that is easy to implement, even in low-capacity laboratories. This approach requires no preliminary serotyping. Second, when more precise discrimination is required, the spacer content can then be investigated by Sanger sequencing of the PCR products, which has the additional advantage of facilitating the detection of new putative spacers. However, once the spacer diversity within a serotype is known (i.e., after the analysis of a representative collection of isolates of this serotype), higher throughput is required for daily surveillance. We have developed a Luminex®-based approach that is suitable for serotype Typhimurium, which accounts for 50% of all cases of human salmonellosis in France and is one of the two major serotypes worldwide. PFGE is currently recommended for the real-time surveillance of this serotype, but is technically demanding and poorly standardized in many laboratories. It is therefore difficult to use PFGE in many countries in which a single reference laboratory processes a large number of isolates. The CRISPOL assay developed here covers both serotype Typhimurium and its emerging monophasic variant. It provides an excellent alternative to PFGE, being cheaper, less technically demanding and yielding data that are easy to interpret and exchange. An approach based on the initial use of the CRISPOL assay, followed by MLVA for genetically homogeneous populations, such as the DT104 clone (CT21) or the emerging monophasic strain (CT1) would be highly effective. However, due to limitations in the number of beads that can be mixed (500 for the latest Luminex® platform), the universal use of a Luminex®-based approach is not possible (the *Salmonella* serotypes analyzed to date include 3,800 different spacers). Whole-genome sequencing (WGS) is a possible alternative, provided costs and analysis times can be decreased. WGS could be customized to focus exclusively on the two CRISPR sequences. The known spacer sequences would be extracted and compared with the contents of a CRISPR/serotype database. Another alternative would involve the use of a microarray approach based on DNA oligonucleotides corresponding to highly informative spacers. In the meantime, subtyping applications remain to be developed for the most epidemiologically important serotypes or MLST groups, such as Enteritidis and Newport. For this purpose, it should be straightforward to apply the strategy presented here for serotype Typhimurium.

Clearly, there is also a need to extend the serotype coverage of the spacer content inventory, as only the 130 most important serotypes have been investigated so far. We hope to capture all the diversity of *Salmonella* (>2,500 serotypes) in the next 10 years, and the CRISPR/serotype dictionary available from our open-access website will be updated accordingly. This web tool can be used to extract and identify spacers from a submitted DNA sequence and for comparisons with a well curated database (i.e., containing accurately serotyped isolates). The application of this tool to CRISPR sequences identified as corresponding to Enteritidis isolates by Liu *et al.* [27], showed that 10 of the 27 considered actually corresponded to Typhimurium (EST21, EST22), Infantis (EST17), Kentucky (EST23), and Heidelberg (EST15), rather than Enteritidis. All these discrepancies related to isolates obtained from a local diagnostic laboratory and not from the reference laboratory participating in the study, which suggests that serotyping errors were the cause.

In conclusion, we have demonstrated that CRISPR is a powerful method suitable for use in the molecular typing and subtyping of *Salmonella* isolates. We believe that, given its combined advantages, CRISPR strain characterization is an excellent potential alternative to both serotyping and PFGE, the current gold standard methods. Given the rapidity of this method, in particular, it should have a major impact on surveillance and outbreak investigation and is likely to be of benefit to public health.

## Materials and Methods

### *Salmonella* Strains and Isolates

In the first part of this study (spacer content inventory and comparison with current typing and subtyping methods), we used 744 *Salmonella* reference strains or isolates belonging to 130 serotypes (including those most frequently identified in human and food products). These serotypes belonged to two species of the *Salmonella* genus: *S. enterica* and *S. bongori* and the six subspecies of *S. enterica*: *enterica*, *salamae*, *arizonae*, *diarizonae*, *indica* and *houtenae* (Tables S2 and S4). The *Salmonella* serotype reference strains were obtained from the World Health Organization Collaborative Center for Reference and Research on *Salmonella* (WHOCC-Salm). Most of the isolates were from the French National Reference Center for *Salmonella* (FNRC-Salm). Both these centers are located at the Institut Pasteur, Paris. Other strain and isolate providers are acknowledged at the end of the manuscript. The strains and isolates studied were obtained from around the world, between 1885 and 2010. Larger subsets of isolates from prevalent serotypes were assembled to reflect as accurately as possible the diversity of these populations: Typhimurium or its monophasic variant with antigenic formula 1,4,[5],12:i:- (n = 150), Enteritidis (n = 187), polyphyletic serotypes such as Newport (n = 21) and Paratyphi B (n = 36), and serotypes with the antigenic formula 6,7:c:1,5 (n = 34), or clinically important serotypes, such as Typhi (n = 20) and Paratyphi A (n = 14). This test population was generally well defined in terms of its epidemiological context, antimicrobial resistance phenotype, phage type, PFGE type, MLVA type, haplotype and MLST type, as determined by methods described elsewhere [9], [32], [33], [40], [41].

In the second part of the study, we validated the CRISPOL method on 150 serotype Typhimurium or 1,4,[5],12:i:- isolates for which both CRISPR loci were sequenced. The method was then applied to a collection of 1,900 isolates from the WHOCC-Salm and from the FNRC-Salm, including all isolates received by the FNRC-Salm from January 1 2010 to July 4 2010 (n = 1,131 isolates from 1,084 patients).

### Inventory of the Spacer Content of 744 *Salmonella* Strains and Isolates and 39 Genomes of 130 Serotypes

#### In silico analysis

We analyzed CRISPR spacer content in 39 full genome sequences of *S. enterica* and *S. bongori* (Table 1). Regions containing CRISPR sequences were identified by a blast (ncbi) search of the 29 bp DR consensus sequence of *S. enterica* serotype Typhi strain Ty2 (5′-CGGTTTATCCCCGCTGGCGCGGGGAACAC-3′) [14]. Regions downstream from the *iap* gene and upstream from the *ygcF* gene were downloaded and the spacer-DR units of each CRISPR locus were extracted manually.

#### DNA extraction

Total DNA was extracted with the InstaGene matrix (BioRad, Marnes la Coquette, France) or the Wizard kit (Promega, Madison, WI, USA) in accordance with the manufacturer’s recommendations.

#### PCR and sequencing of the CRISPR1 and CRISPR2 loci

Oligonucleotide primers for amplification of the CRISPR1 and CRISPR2 loci from all *Salmonella* spp. were designed on the basis of consensus alignments of the available *Salmonella* genomes (Tables 1 and 2). The CRISPR1 locus was amplified with the forward A1 primer (binding 74 bp upstream from the CRISPR1 of serotype Typhimurium strain LT2) and the reverse primer A2 (binding 130 bp downstream). Alternative reverse primers, such as A3 to A7, were required for some isolates. The CRISPR2 locus was amplified with the forward primer B1 (binding 110 bp upstream from CRISPR2) and the reverse primers B2 and B3 (binding 45 bp and 324 bp downstream from the CRISPR2 locus of strain LT2, respectively). The B3 primer was designed because no region homologous to B2 was found in subsp. *diarizonae*. The primers were synthesized by MWG-Biotech (Ebersberg, Germany). Single-locus amplifications were performed in a total volume of 50 µl containing DNA (2.5 µl from InstaGene matrix or 2 µl diluted 10-fold from Wizard), primers (10 pmol each), deoxynucleoside triphosphate (100 µM), *Taq* DNA polymerase (0.85 U of GoTaq Flexi DNA polymerase; Promega) and its buffer, MgCl_2_ (1.5 mM) and dimethylsulfoxide (5%). The cycling conditions were as follows: 2 min for denaturation at 94°C (1 cycle), followed by 35 cycles of 1 min at 94°C for denaturation, 1 min at 59°C (61°C when using the A1-A4 pair) for annealing, and 90 s at 72°C for polymerization, followed by an additional 10 min at 72°C for extension.

The entire region spanning both CRISPR loci was amplified with primers A1 and B3. For this purpose, DNA was extracted with the Promega Wizard kit and PCR was carried out with the Expand Long Template PCR System kit (Roche).

Both strands of purified amplicons were sequenced with Big Dye Terminator version 3.1 (Applied Biosystems, Foster City, CA) on an ABI 3730XL apparatus (Applied Biosystems).

BioNumerics 6.5 software (Applied Maths, Sint-Martens-Latem, Belgium) was used to analyze nucleotide sequences.

### Development of Web-accessible Tools and CRISPR/serotype Dictionary

We have developed a web tool for the creation and storage of catalogues of spacers and DR variants. This “Institut Pasteur CRISPR database for *Salmonella*” can be queried online at http://www.pasteur.fr/recherche/genopole/PF8/crispr/CRISPRDB.html. The content of the catalogue is used to identify known spacers and DRs in a submitted DNA sequence, which is coded into a succession of DR and spacer identifiers by the query “**Search spacers composition for query**”. If part of the sequence has no exact matches in the DR and spacers dictionary, a blast query can then be used to obtain the nearest match (“**Blast unknown spacer sequence against dictionary**”), to identify new spacers or new DR variants. Isolates analyzed at the FNRC-Salm and coded as spacer-DR arrays within the CRISPR/serotype dictionary can be downloaded with the “**Browse spacers composition for the published strains**” query. .

#### Spacer nomenclature

The spacer names start with a three- to four-letter prefix indicating the serotype from which the spacer was extracted for the first time. The suffix B indicates spacers found in the CRISPR2 locus. Spacers were numbered consecutively in order of discovery. The start of spacer arrays is described, starting downstream from the *iap* gene. SNP or VNTR variants are denoted as “var” (e.g., EntB0var1, STM18var2).

#### Calculation of discrimination indices

The discriminatory abilities of the CRISPR, PFGE and phage typing methods were assessed by calculating Simpson’s index of diversity (D value), as previously described [42].

#### Statistic analysis

The mean number and standard deviation of the spacers in the CRISPR loci were calculated with Excel (Microsoft).

#### Nucleotide sequence accession numbers

The nucleotide sequences of the CRISPR loci have been assigned GenBank accession numbers JF724159 to JF725640.

### Development of a High-throughput Subtyping Method (CRISPOL) for Serotype Typhimurium

#### DNA extraction

We increased the throughput of this method by using thermolysates as the DNA template. Briefly, we suspended a 10 µl loop of bacteria in 200 µl of molecular biology-grade water. The suspension was vortexed for 10s, incubated at 95°C for 10 min and then centrifuged for 5 minutes at 13000 rpm in a Jouan A14 centrifuge. The supernatant was transferred to a 1.5 ml microtube and stored at −20°C until use.

#### PCR amplification

We followed the strategy used for *Mycobacterium* spoligotyping, based on the use of two primers hybridizing to the DRs in opposite directions, one of which was 5′-biotinylated [17]. The primers were DRSTMA (5′-CCGCTGGCGCGGGGAACA-3′) and DRSTMB (5′Biot-CGCCAGCGGGGATAAACC-3′). Amplifications were carried out in a volume of 50 µl containing 1 µl of thermolysate (or 1 µl of molecular biology-grade water for the blank), primers (50 pmol each), deoxynucleoside triphosphate (200 µM), *Taq* DNA polymerase (0.85 U of Go *Taq*; Promega) and its buffer, MgCl_2_ (1.5 mM). The cycling conditions were as follows: 2 min at 95°C for initial denaturation, followed by 20 cycles of 1 min at 95°C for denaturation, 30 s at 59°C for annealing and 15 s at 72°C for polymerization. The PCR products were checked by electrophoresis in 1.2% agarose gels and were stored at −20°C for no more than three days before use in the Luminex® assay.

#### Probes and microbead coupling

We designed 72 spacer-derived oligonucleotide probes (including four SNP-variant probes) of between 25 and 32 nucleotides in size (Table 6). These probes were synthesized by Eurogentec (Angers, France), with a 5′ terminal amino group modification, using a 12-carbon spacer linker. The 72 Luminex xMap microbeads (L100-C129 to L100-C200) were coupled to the 72 probes, as previously described [43]. Each type of coupled microbead was resuspended in 1 × TE (10 mM Tris, 1 mM EDTA, pH 8) at a final concentration of approximately 50,000 microbeads per µl. We then combined equal volumes of each type of coupled microbead in Protein LoBind tubes (Eppendorf, Hamburg, Germany). The mixture was stored in the dark at 4°C before and after use.

#### Hybridization

Hybridization was performed in a polycarbonate plate with 96 conical wells (Corning, Corning, NY, USA), to which we added 10 µl of PCR product, 7 µl of 1 × TE and 33 µl of probe-coupled microbeads diluted in 1.5 × TMAC buffer (5 M tetramethyl ammonium chloride [Sigma, St. Louis, MO, USA], 20% Sarkosyl, 1 M Tris-HCl [pH 8.0], 0.5 M EDTA [pH 8.0]) to a final concentration of approximately 75 microbeads per µl for each type of coupled microbead. The plates were sealed with adhesive PCR film (ABgene, Epsom, UK) and heated to 94°C for 3 min for initial denaturation, followed by hybridization at 59°C for 20 min. The plate was centrifuged at 4,000 g for 3 min and the supernatant was carefully discarded. A reporter mix consisting of 90 µl of streptavidin-R-phycoerythrin (1.25 µg/ml in 1 × TMAC; Invitrogen, Carlsbad, CA, USA) was added to each well and the microbead pellet was resuspended. The microplate was then incubated for 5 minutes and analyzed on the Luminex® platform.

#### Analysis on the Luminex platform

The microplate was analyzed in a Luminex® 200 system at a temperature of 50°C. Analyses were based on counts for 50 beads per set.

#### Data analysis

Four strains (SARA8, 81-784, 02-7015 and 07-1777) with a known spacer content covering all the spacers in the assay and a blank (in which DNA was replaced with water) were analyzed in each experiment. For all probes except pSTMB26, relative fluorescence unit values were corrected by subtracting the value for the blank. In the case of negative corrected values, an arbitrary value of 25 was attributed. For STMB26, values were corrected by subtracting those for 07-1777, due to a weak cross-reaction observed in emerging monophasic variant strains (07-1777 being a monophasic variant isolate). A cutoff value five times higher than the corrected value was defined. For pSTM03, pSTM07, pSTM12 and pSTMB9 probes and their SNP variant probes pSTM03var1, pSTM07var2, pSTM12var1, and pSTMB9var1, assignment to the wild-type spacer or its SNP variant was based on the ratio of crude values for each probe. If the wild-type probe/SNP variant probe ratio was >1.1, then the wild-type spacer was attributed to the isolate. If the wild-type probe/SNP variant probe ratio was <0.9, then the SNP variant spacer was attributed to the isolate. An R tool was developed to automate the analysis. For each strain an allelic pattern, referred to as the CRISPOL type (CT), consisting of the presence or absence of the 68 ordered CRISPR1, then CRISPR2 probes, followed by the four SNP-variant probes, was generated. Data were incorporated into a dedicated CRISPOL database with BioNumerics® software. A provisional number was assigned to each strain with a new CT, (e.g., CT62prov) until both CRISPR regions had been sequenced, to check for consistency with the CT and for an absence of new spacers.

## Acknowledgments

We thank K. Sanderson (Salmonella Genetic Stock Centre, University of Calgary, Canada), A. Brisabois (Anses, Maisons-Alfort, France), M. Mikoleit (Centers for Disease Control and Prevention, Atlanta, USA), C. Bizet (Collection de l’Institut Pasteur, Paris, France), L. Bossi (Centre National de la Recherche Scientifique, Gif-sur-Yvette, France), L. Ward (Health Protection Agency, Colindale, United Kingdom), J.D. Perrier-Gros-Claude (Institut Pasteur de Dakar, Dakar, Senegal), J. Lassen (Norwegian Institute of Public Health, Oslo, Norway), A. Miko (Federal Institute for Risk Assessment (BfR), Berlin, Germany), C.H. Chiu (Chang Gung Children’s Hospital, Taoyuan, Taiwan), and R. Hendriksen (Technical University of Denmark, Lyngby, Denmark) for providing some of the strains studied. We thank F. Topin (Luminex B.V., Oosterhout, The Netherlands) for his support.

## Supporting Information

Figure S1 *S. enterica* serotype Typhimurium spacers of non classical length.
(DOC)

Figure S2 Distribution of median fluorescence intensity (MFI) values for each probe in a typical CRISPOL experiment with 65 isolates.
(DOC)

Table S1 Direct repeats in the 39 available genomes of *Salmonella* spp.
(DOC)

Table S2 Characteristics, CRISPR alleles, and spacer sequences of the 783 *Salmonella* strains, isolates and genomes studied.
(XLS)

Table S3 DNA sequences of the inventoried spacers.
(XLS)

Table S4 Number of spacers per *Salmonella* serotype.
(DOC)

Table S5 Comparison of CRISPR2 spacer content with the population structure of *S. enterica* serotype Newport as assessed by MLST.
(DOC)

Table S6 CRISPR2 spacer content in various O:9 and O:2 serotypes.
(DOC)

Table S7 Characteristics, PFGE types, MLVA types, phage types, CRISPR alleles, and CRISPOL types of the 158 *S. enterica* serotype Typhimurium strains and isolates (including the monophasic variant) studied.
(XLS)

Table S8 Epidemiological concordance of CRISPR spacer content for separate outbreaks due to serotype Enteritidis.
(DOC)

Table S9 Probe responses in the CRISPOL assay for triplicates of 5 individual strains or isolates.
(DOC)
