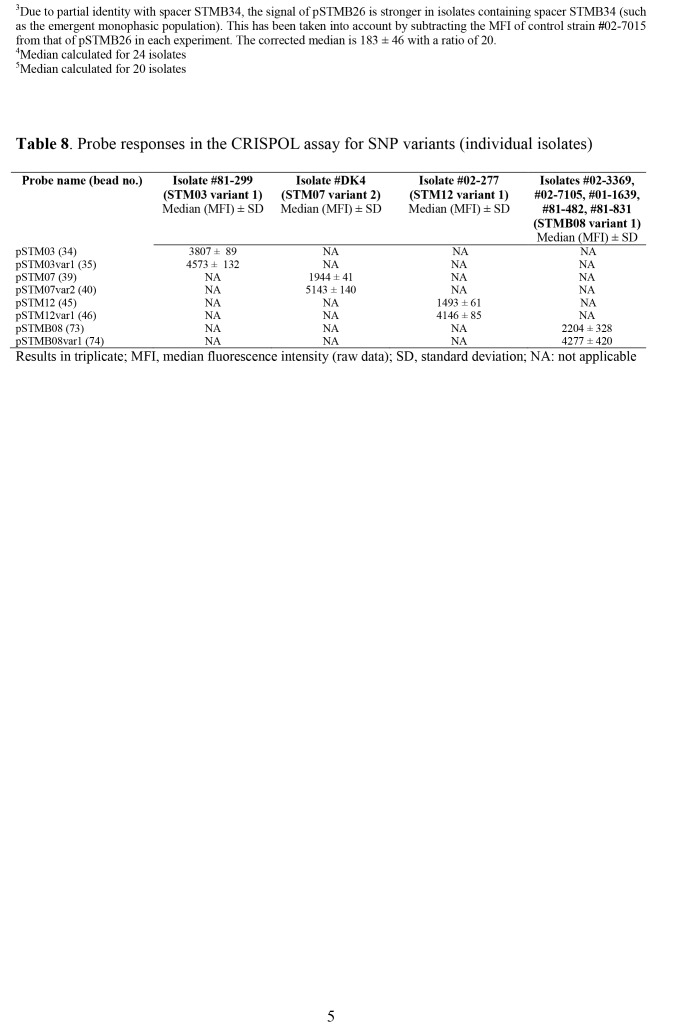# Correction: CRISPR Typing and Subtyping for Improved Laboratory Surveillance of *Salmonella* Infections

**DOI:** 10.1371/annotation/e79cea9a-6716-4519-9e96-31b17bf6a4fb

**Published:** 2012-11-07

**Authors:** Laëtitia Fabre, Jian Zhang, Ghislaine Guigon, Simon Le Hello, Véronique Guibert, Marie Accou-Demartin, Saïana de Romans, Catherine Lim, Chrystelle Roux, Virginie Passet, Laure Diancourt, Martine Guibourdenche, Sylvie Issenhuth-Jeanjean, Mark Achtman, Sylvain Brisse, Christophe Sola, François-Xavier Weill

There were errors in Tables 1, 3, 5, 7, and 8. Footnote citations were missing from Table 1, 5, and 7. Text was missing from Table 3. There were formatting errors in Table 8. The corrected tables can be viewed here: 

Table 1: 

**Figure:**
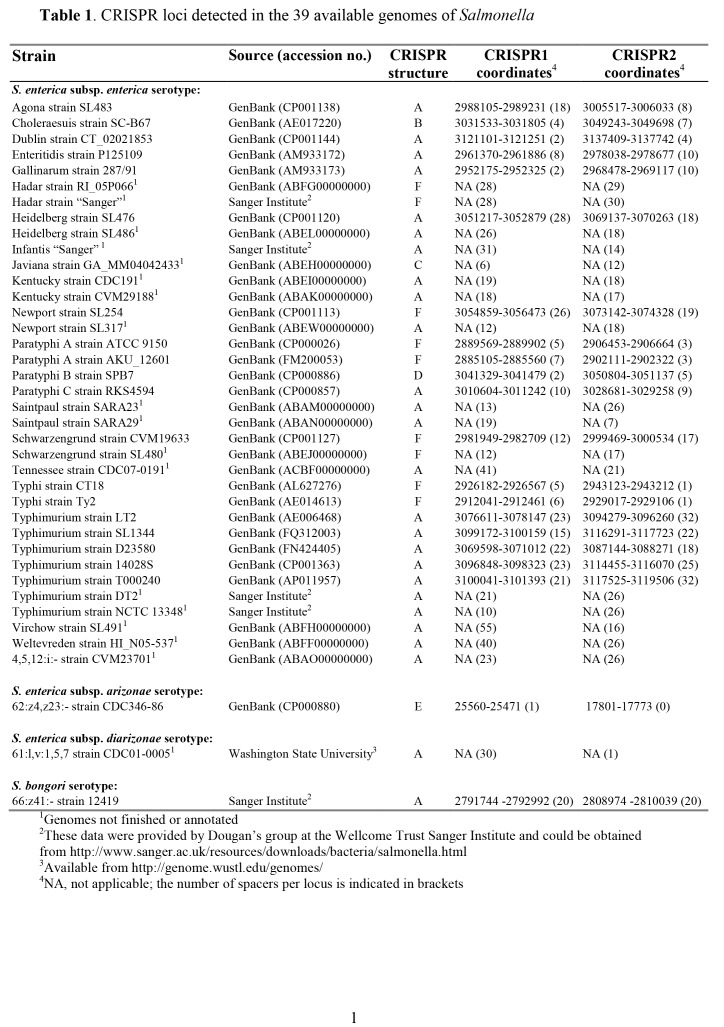


Table 3: 

**Figure:**
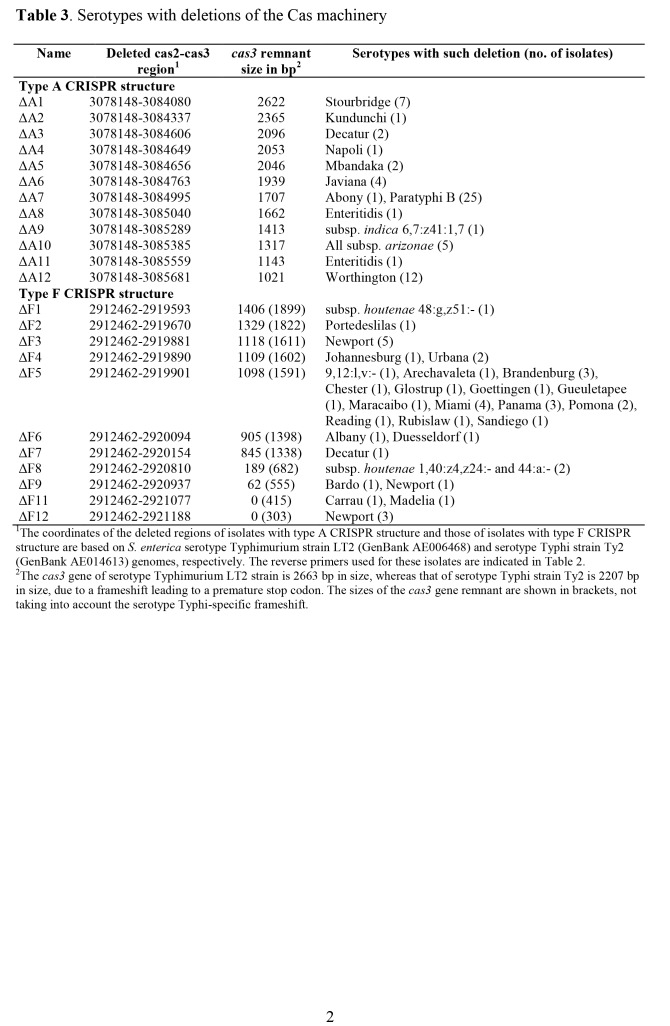


Table 5: 

**Figure:**
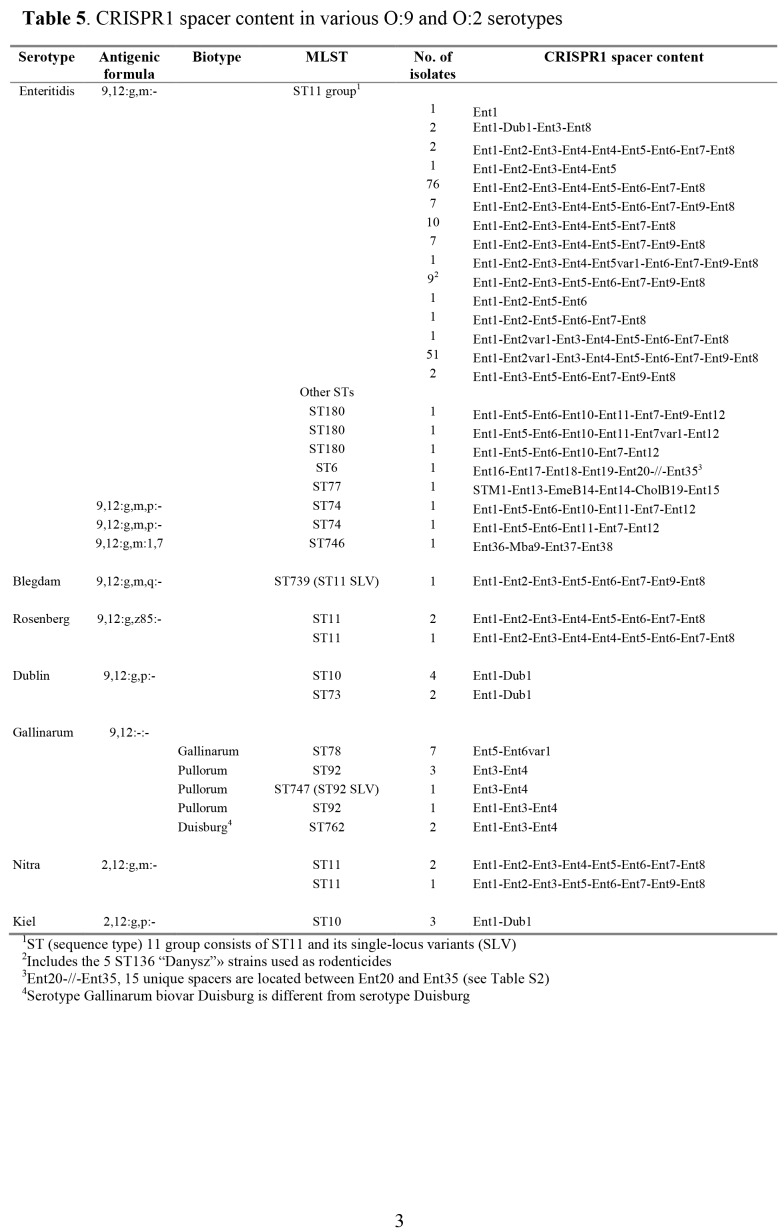


Table 7: 

**Figure:**
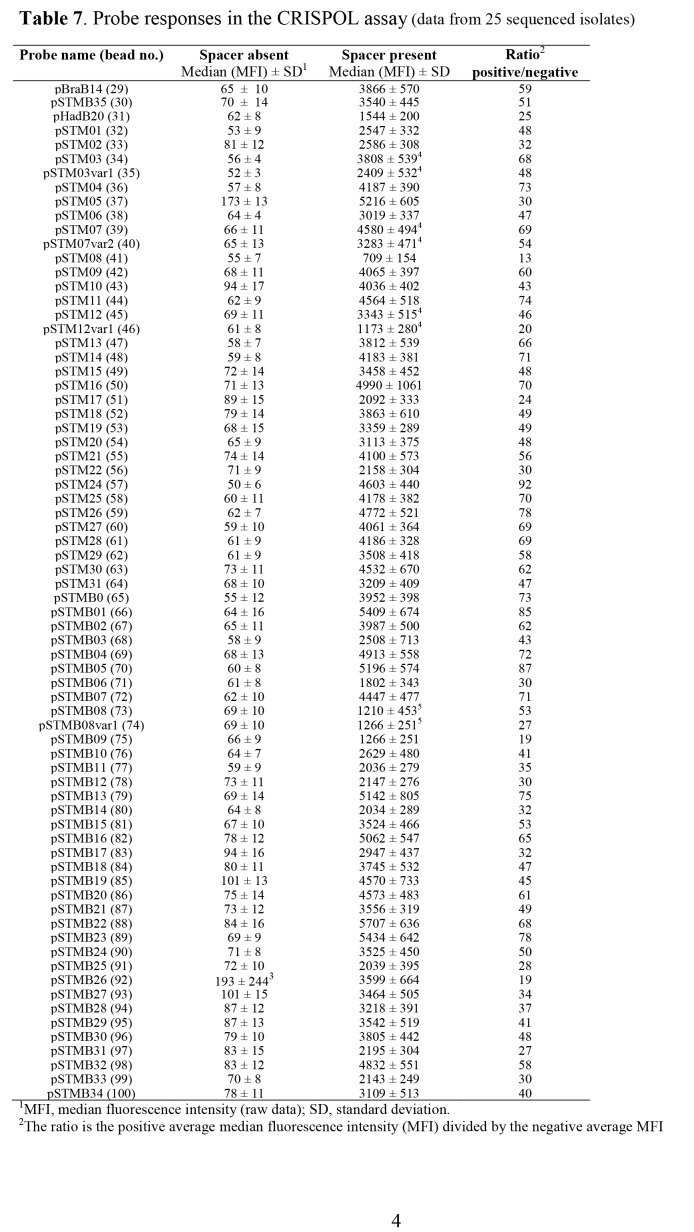


Table 8: 

**Figure:**