# Supplementary material for: CRISPR Typing and Subtyping for Improved Laboratory Surveillance of Salmonella Infections
Source: PLoS One. 2012 May 18;7(5):e36995. doi: 10.1371/journal.pone.0036995 (PMC3356390; doi:10.1371/journal.pone.0036995)
Supplement: Figure S1 — S. enterica serotype Typhimurium spacers of non classical length. (DOC) [file pone.0036995.s001.doc]

**
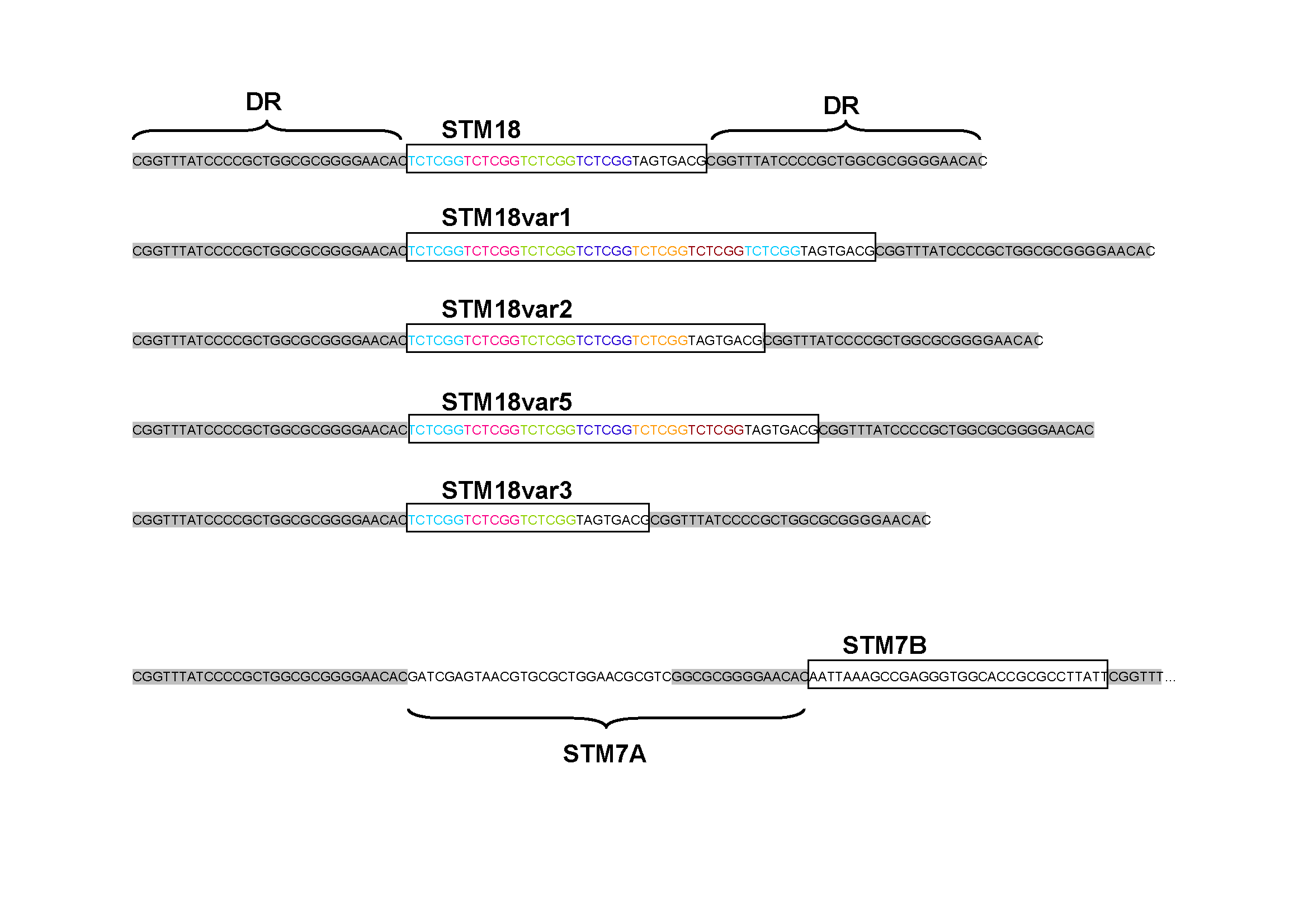
**

**Figure S1.** *S. enterica* serotype Typhimurium spacers of non classical length.

STM18 variants differ from STM18 by having a variable number of tandem repeats (TCTCGG motif). STM18 (33bp) is the most common (found in 14/158 isolates), but each of the variants has been found in at least one strain. Upstream from STM7B, there was a 28-nucleotide variable sequence followed by a remnant of DR (14 nucleotides from the 3’ end). This hybrid unit (spacer-DR), STM7A, may have been generated accidentally during the spacer acquisition process, possibly due to homology between the 3’end of the initial spacer sequence and the DR.
