# Supplementary material for: CRISPR Typing and Subtyping for Improved Laboratory Surveillance of Salmonella Infections
Source: PLoS One. 2012 May 18;7(5):e36995. doi: 10.1371/journal.pone.0036995 (PMC3356390; doi:10.1371/journal.pone.0036995)
Supplement: Figure S2 — Distribution of median fluorescence intensity (MFI) values for each probe in a typical CRISPOL experiment with 65 isolates. (DOC) [file pone.0036995.s002.doc]

**
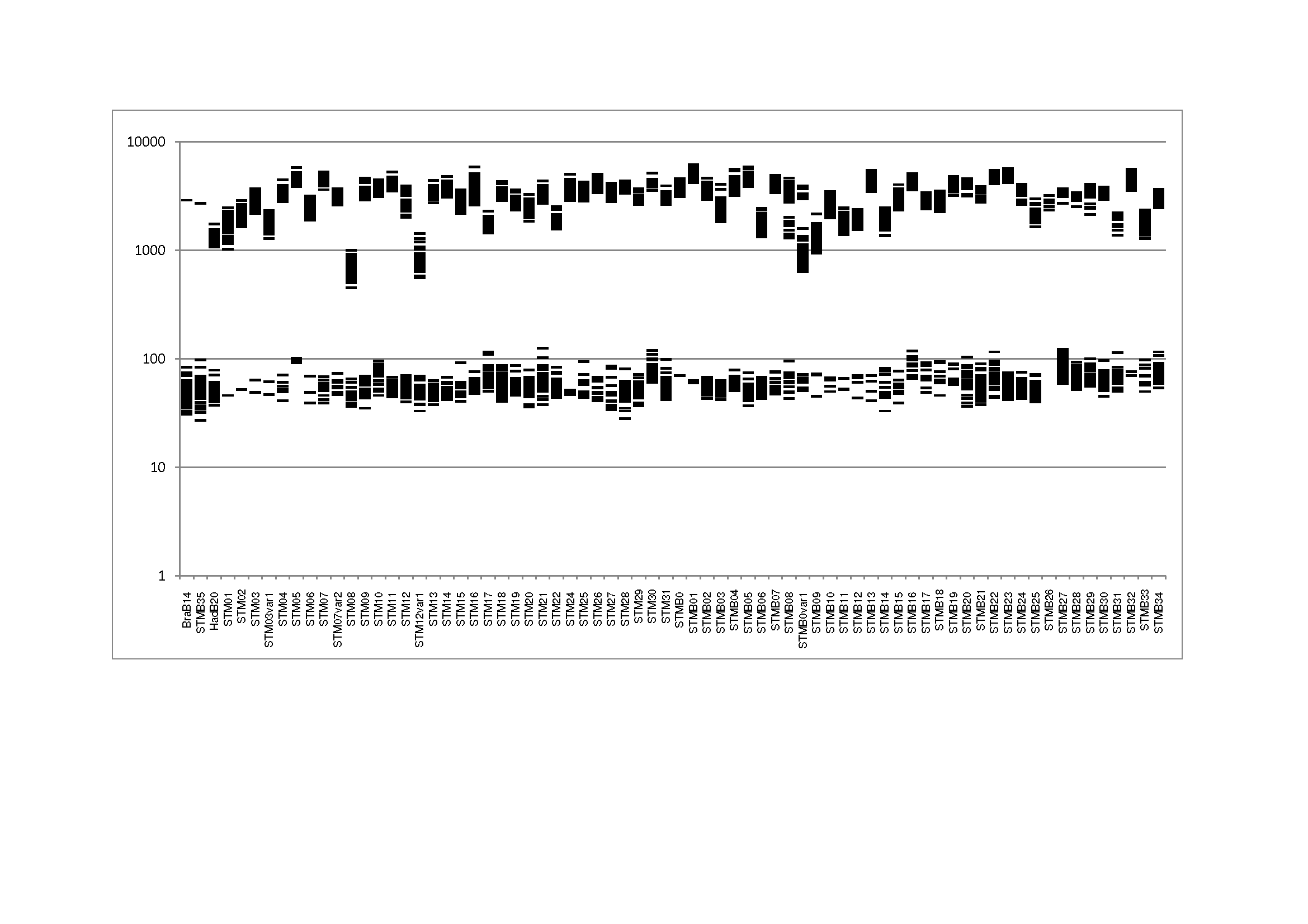
**

**Figure S2.** Distribution of median fluorescence intensity (MFI) values for each probe in a typical CRISPOL experiment with 65 isolates.

The corrected MFI values (MFI of sample minus MFI of blank for all probes, except for pSTMB26, for which the values are MFI sample minus the MFI of control strain **#**02-7015 – see text) are shown.
