# Supplementary material for: CRISPR Typing and Subtyping for Improved Laboratory Surveillance of Salmonella Infections
Source: PLoS One. 2012 May 18;7(5):e36995. doi: 10.1371/journal.pone.0036995 (PMC3356390; doi:10.1371/journal.pone.0036995)
Supplement: Table S1 — Direct repeats in the 39 available genomes of Salmonella spp. (DOC) [file pone.0036995.s003.doc]

**Table S1**. Direct repeats in the 39 available genomes of *Salmonella* spp.

| **Direct Repeat Name** | **Sequence 5'-3'** | **n** | **Percentage** |
| --- | --- | --- | --- |
| DR | CGGTTTATCCCCGCTGGCGCGGGGAACAC | 1123 | 79,8 |
| DR6 | CGGTTTATCCCCGCTAGCGCGGGGAACAC | 42 | 3 |
| DR1C | ACGGCTATCCTTGTTGGCGCGGGGAACAC | 24 | 1,7 |
| DR47 | CGGTTTATCCCCGCTGGCGCGGGGAATAC | 16 | 1,1 |
| DR28 | AGGTTTATCCCCGCTGGCGCGGGGAACAC | 13 | <1 |
| DR8 | CGGTTTATCCCTGCTGGCGCGGGGAACAC | 13 | <1 |
| DR13 | CGGTTTATCCCCGCTGGCACGGGGAACAC | 12 | <1 |
| DR14 | CGGTTTATCCCCGCTGGCGCGGGAAACAC | 12 | <1 |
| DR18 | CAGTTTATCCCCGCTGGCGCGGGGAACAC | 11 | <1 |
| DR12 | GTGTTTATCCCCGCTGACGCGGGGAACAT | 10 | <1 |
| DR22 | GTGTTTATCCCCGCTGGCGCGGGGAACAT | 10 | <1 |
| DR9 | CGGTTTATCCCCGCTGACGCGGGGAACAC | 10 | <1 |
| DR4 | CGGCTTATCCCCGCTGGCGCGGGGAACAC | 9 | <1 |
| DR1A1 | GTGTTTATCCCCGCTGGCGCGGGGAACAC | 8 | <1 |
| DR1B | GTGTTTATCCCCGCTGACGCGGGGAACAC | 8 | <1 |
| DR20 | CGGTTTATCCCCGCTAGCGCGGGGAACAT | 8 | <1 |
| DR48 | CGGTTTATCCCCGCTGGCGCGGGGAGCAC | 8 | <1 |
| DR5 | CGATTTATCCCTGCTGGCGCGGGGAACAC | 7 | <1 |
| DR21 | ACGGCTATCCCCGCTGGCGCGGGGAACAC | 5 | <1 |
| DR3 | GGGTTTATCCCCGCTGGCGCGGGGAACAC | 5 | <1 |
| DR33 | ACGGCTATCCCCGCTGACGCGGGGAACAC | 4 | <1 |
| DR85 | CGGTTTATCCCCGCTGACGCGGGGAACAT | 4 | <1 |
| DR11 | CGGTTTATCCCCGCTGGCGCGGGGAACAT | 3 | <1 |
| DR42 | CGGTTTATCCCCGCTGGCGAGGGGAACAC | 3 | <1 |
| DR64 | CGGTTTATCCCCGCTGGCGCGGGGAT | 3 | <1 |
| DR15 | CGGTTTATCCCCGCTGGTGCGGGGAACAC | 2 | <1 |
| DR1P | CGGTTATCCCCGCTGGCGCGGGGAACAC | 2 | <1 |
| DR2 | CGATTTATCCCCGCTGGCGCGGGGAACAC | 2 | <1 |
| DR27 | ACGGCTATCCTTGTTGACGTGGGGAATAC | 2 | <1 |
| DR49 | CAGTTTATCCCCGCTAGCGCGGGGAACAC | 2 | <1 |
| DR67 | CGGTTTATCCCCGCTGGCGCGGAGAACAC | 2 | <1 |
| DR76 | CGGTTTATCCCCGCTGGCGCGAGGAACAC | 2 | <1 |
| DR84 | CGGTTTACCCCCGCTGACGCGGGGAACAT | 2 | <1 |
| DR86 | CGGTTTATCCCCGCTGACGCGGGGGACAT | 2 | <1 |
| DR122 | CGGTTTATCCCCTCTGGCGCGGGGAACAC | 1 | <1 |
| DR123 | CGGTCTATCCCCGCTGGCGCGGGGAACAC | 1 | <1 |
| DR1bon | CAGTAAAAGCCCCGCTGGCGCGGGGAACAC | 1 | <1 |
| DR1IIIb | TAGCTTATCCCCGCTGACGCGGGGAACAC | 1 | <1 |
| DR23 | CGGTTTATCTCCGCTGGCGCGGGGAACAC | 1 | <1 |
| DR25 | GGGTTTATCCCCGCTGGCGCAGGGAACAC | 1 | <1 |
| DR26 | CGGTTTATCCCCGCTGGCGCTGGGAACAC | 1 | <1 |
| DR30 | CGGTTTATCCCCGCTAGCGCGGGGAAGAC | 1 | <1 |
| DR31 | CGGTTTATCCCCGCTGCGCGGGGAACAC | 1 | <1 |
| DR32 | CGGTTTATCCCCGCTGGCGCGGTGAGAAAC | 1 | <1 |
| DR45 | GGGTTTATCCCCGCTAGCGCGGGGAACAC | 1 | <1 |
| DR55 | CGGTTTATCCCCGCTCGCGCGGGGAACAC | 1 | <1 |
| DR65 | TAGTTTATCCCCGCTGACCCGGGGAACAT | 1 | <1 |
| DR68 | CGGTTTATCCCCGCTAACGCGTGGAACAT | 1 | <1 |
| DR69 | CGACTTATCCCCGCTGGCGCGGGGAACAC | 1 | <1 |
| DR75 | CGGTTTATCCCCGCGGGCGTGGGGAACAC | 1 | <1 |
| DR82 | CGGTTTATCCCCGCTGCCGCGGGGAACAC | 1 | <1 |
| DR99 | CGGTTTATCCCCGCTGGCGCGGGGAACCC | 1 | <1 |
