# Supplementary material for: CRISPR Typing and Subtyping for Improved Laboratory Surveillance of Salmonella Infections
Source: PLoS One. 2012 May 18;7(5):e36995. doi: 10.1371/journal.pone.0036995 (PMC3356390; doi:10.1371/journal.pone.0036995)
Supplement: Table S4 — Number of spacers per Salmonella serotype. (DOC) [file pone.0036995.s006.doc]

**Table S4**. Number of spacers per *Salmonella* serotype

| **Species** | **Subspecies** | **Serotype** | **Antigenic formula** | **Number of isolates** | **CRISPR 1 locus** | | **CRISPR 2 locus** |
| --- | --- | --- | --- | --- | --- | --- | --- |
|  |  |  |  |  | **Primers for PCR** | **Number of spacers** | **Number of spacers** |
| *S. enterica* | *enterica* | **Group O:2 (A)** |  |  |  |  |  |
|  |  | Paratyphi A | 1,2,12:a:[1,5] | 14^2^ | A1-A2 | 5-7 | 3 |
|  |  | Nitra | 2,12:g,m:- | 3 | A1-A2 | 8 | 8-10 |
|  |  | Kiel | 1,2,12: g,p:- | 3 | A1-A2 | 2 | 3-5 |
|  |  | **Group O:4 (B)** |  |  |  |  |  |
|  |  | Kisangani | 1,4,[5],12:a:1,2 | 1 | A1-A2 | 16 | 24 |
|  |  | Hessarek | 4,12,[27]:a:1,5 | 1 | A1-A2 | 8 | 6 |
|  |  | Arechavaleta | 4,[5],12:a:1,7 | 1 | A1-A4 | 1 | 7 |
|  |  | Bispebjerg | 1,4,[5],12:a:e,n,x | 1 | A1-A2 | 19 | 10 |
|  |  | Fulica | 4,[5],12:a:- | 1 | A1-A2 | 1 | 5 |
|  |  | Paratyphi B | 1,4,[5],12:b:1,2 | 25^1^ | A1-A3 | 1-4 | 2-6 |
|  |  |  |  | 11 | A1-A2 | 10-24 | 1-20 |
|  |  | Limete | 1,4,12,[27]:b:1,5 | 2 | A1-A2 | 40-41 | 16 |
|  |  | Canada | 4,12,[27]:b:1,6 | 2 | A1-A2 | 10-26 | 12-28 |
|  |  | Tripoli | 1,4,12,27:b:z_6_ | 1 | A1-A2 | 34 | 20 |
|  |  | Abony | 1,4,[5],12,27:b:e,n,x | 2 | A1-A3 | 5-7 | 1-10 |
|  |  | Schleissheim | 4,12,27:b:- | 1 | A1-A2 | 23 | 10 |
|  |  | Abortusovis | 4,12:c:1,6 | 2 | A1-A2 | 2-19 | 2-5 |
|  |  | Bissau | 4,12:c:e,,n,x | 1 | A1-A2 | 48 | 36 |
|  |  | Schwarzengrund | 1,4,12,[27]:d:1,7 | 5^2^ | A1-A2 | 12-15 | 17 |
|  |  | Saintpaul | 1,4,[5],12:e,h:1,2 | 5^2^ | A1-A2 | 13-27 | 7-26 |
|  |  | Reading | 1,4,[5],12:e,h:1,5 | 1 | A1-A4 | 1 | 1 |
|  |  | Chester | 1,4,[5],12:e,h:e,n,x | 1 | A1-A4 | 1 | 1 |
|  |  | Sandiego | 1,4,[5],12:e,h:e,n,z_15_ | 1 | A1-A4 | 14 | 6 |
|  |  | Derby | 1,4,[5],12:f,g:[1,2] | 7 | A1-A2 | 18-32 | 10-45 |
|  |  | Agona | 1,4,[5],12:f,g,s:[1,2] | 14^1^ | A1-A2 | 13-46 | 7-25 |
|  |  | Typhimurium | 1,4,[5],12:i:1,2 | 143^3^ | A1-A2 | 6-31 | 4-40 |
|  |  | 1,4,[5],12:i:- | 1,4,[5],12:i:- | 15^1^ | A1-A2 | 8-31 | 21-30 |
|  |  | Bredeney | 1,4,12,27:l,v:1,7 | 1 | A1-A2 | 15 | 21 |
|  |  | Brandenburg | 4,[5],12:l,v:e,n,z_15_ | 3 | A1-A4 | 1 | 6-7 |
|  |  | Kundunchi | 1,4,[5],12,[27]:l,[z_13_],[z_28_]:1,2 | 1 | A1-A3 | 1 | 9 |
|  |  | Indiana | 1,4,12:z:1,7 | 3 | A1-A2 | 17-22 | 15-17 |
|  |  | Heidelberg | 1,4,[5],12:r:1,2 | 7^2^ | A1-A2 | 18-29 | 18-34 |
|  |  | Stanleyville | 1,4,[5],12,[27]:z_4_,z_23_:[1,2] | 1 | A1-A2 | 17 | 19 |
|  |  | Abortusequi | 4,12:-:e,n,x | 3 | A1-A2 | 2 | 10 |
|  |  | **Group O:7 (C1)** |  |  |  |  |  |
|  |  | Sanjuan | 6,7:a1,5 | 1 | A1-A2 | 54 | 16 |
|  |  | Paratyphi C | 6,7,[Vi]:c:1,5 | 5^1^ | A1-A2 | 9-10 | 9-11 |
|  |  | Choleraesuis | 6,7:c:1,5 | 20^1^ | A1-A2 | 3-27 | 4-42 |
|  |  |  |  | 2 | A1-A3 | 5 | 9 |
|  |  |  |  | 1 | A1-A8 | 5 | 18 |
|  |  | Typhisuis | 6,7:c:1,5 | 6 | A1-A2 | 6-8 | 1 |
|  |  | Lomita | 6,7:e,h:1,5 | 1 | A1-A2 | 47 | 4 |
|  |  | Braenderup | 6,7,14:e,h:e,n,z_15_ | 1 | A1-A2 | 34 | 18 |
|  |  | Montevideo | 6,7,14:g,m,[p],s:[1,2,7] | 18 | A1-A2 | 6-34 | 2-23 |
|  |  | Thompson | 6,7,14:k:1,5 | 1 | A1-A2 | 27 | 19 |
|  |  | Concord | 6,7:l,v:1,2 | 4 | A1-A2 | 14-32 | 26-27 |
|  |  | 6,7:l,v:- |  | 2 | A1-A2 | 31 | 24-27 |
|  |  | Postdam | 6,7,14:l,v:e,n,z_15_ | 1 | A1-A2 | 21 | 12 |
|  |  | Oranienburg | 6,7,14:m,t:[z_57_] | 1 | A1-A2 | 32 | 15 |
|  |  | Virchow | 6,7,14:r:1,2 | 4^1^ | A1-A2 | 31-41 | 16-22 |
|  |  | Infantis | 6,7,14:r:1,5 | 4^1^ | A1-A2 | 29-31 | 14-26 |
|  |  | Mikawasima | 6,7,14:y:e,n,z_15_ | 1 | A1-A2 | 33 | 3 |
|  |  | Mbandaka | 6,7,14:z_10_:e,n,z_15_ | 2 | A1-A2 | 21-124 | 16-42 |
|  |  |  |  | 2 | A1-A3 | 11 | 21 |
|  |  | Tennessee | 6,7,14:z_29_:[1,2,7] | 2^1^ | A1-A2 | 41-63 | 21-23 |
|  |  | **Group O:8 (C2-C3)** |  |  |  |  |  |
|  |  | Stourbridge | 6,8:b:1,6 | 7 | A1-A3 | 10-33 | 5-14 |
|  |  | Muenchen | 6,8:d:1,2:[z_67_] | 1 | A1-A2 | 18 | 2 |
|  |  | Manhattan | 6,8:d:1,5 | 8 | A1-A2 | 9-11 | 2 |
|  |  | Newport | 6,8,20:e,h:1,2:z_67_ | 12^2^ | A1-A2 | 11-26 | 10-21 |
|  |  |  |  | 4 | A1-A5 | 6-30 | 12-20 |
|  |  |  |  | 5 | A1-A7 | 13-27 | 29-30 |
|  |  | Bardo | 8:e,h:1,2 | 1 | A1-A5 | 12 | 22 |
|  |  | Kottbus | 6,8:e,h:1,5 | 3 | A1-A2 | 17-39 | 9-25 |
|  |  | Emek | 8,20:g,m,s:- | 2 | A1-A2 | 6 | 20 |
|  |  | Chincol | 6,8:g,m,[s]:[e,n,x] | 1 | A1-A2 | 11 | 12 |
|  |  | Lindenburg | 6,8:i:1,2 | 1 | A1-A2 | 10 | 15 |
|  |  | Blockley | 6,8:k:1,5 | 1 | A1-A2 | 14 | 29 |
|  |  | Litchfield | 6,8:l,v:1,2 | 1 | A1-A2 | 8 | 7 |
|  |  | Bovismorbificans | 6,8,20:r,[i]:1,5 | 3 | A1-A2 | 22-23 | 15-17 |
|  |  | Kentucky | 8,20:i:z_6_ | 18^2^ | A1-A2 | 13-37 | 17-37 |
|  |  | Altona | 8,20:r,[i]:z_6_ | 2 | A1-A2 | 21-22 | 20-25 |
|  |  | Duesseldorf | 6,8:z_4_,z_24_:- | 1 | A1-A4 | 8 | 7 |
|  |  | Albany | 8,20:z_4_,z_24_:- | 1 | A1-A4 | 7 | 4 |
|  |  | Tallahassee | 6,8:z_4_,z_32_:- | 1 | A1-A2 | 3 | 7 |
|  |  | Istanbul | 8:z_10_:e,n,x | 1 | A1-A2 | 29 | 25 |
|  |  | Hadar | 6,8:z_10_:e,n,x | 5^2^ | A1-A2 | 28 | 29-31 |
|  |  | Glostrup | 6,8:z_10_:e,n,z_15_ | 1 | A1-A4 | 1 | 5 |
|  |  | Aesch | 6,8:z_60_:1,2 | 1 | A1-A2 | 28 | 18 |
|  |  | **Group O:9 (D1)** |  |  |  |  |  |
|  |  | Miami | 1,9,12:a:1,5 | 6 | A1-A2 | 4-9 | 7-18 |
|  |  |  |  | 4 | A1-A4 | 1 | 6 |
|  |  | Sendai | 1,9,12:a:1,5 | 1 | A1-A2 | 3-4 | 1 |
|  |  | Eschberg | 9,12:d:1,7 | 1 | A1-A2 | 35 | 24 |
|  |  | Typhi | 9,12,[Vi]:d:- | 20^2^ | A1-A2 | 2-6 | 1 |
|  |  | Berta | 1,9,12:[f],g,[t]:- | 1 | A1-A2 | 3 | 5 |
|  |  | Enteritidis | 1,9,12:[f],g,m,[p]:[1,7] | 178^1^ | A1-A2 | 1-9 | 4-19 |
|  |  |  |  | 2 | A1-A3 | 4-21 | 13-33 |
|  |  | Gueuletapee | 9,12:g,m,s:- | 1 | A1-A4 | 1 | 11 |
|  |  | Blegdam | 9,12:g,m,q:- | 1 | A1-A2 | 8 | 8 |
|  |  | Dublin | 1,9,12,[Vi]:g,p:- | 6^1^ | A1-A2 | 2 | 4-5 |
|  |  | Rosenberg | 9,12:g,z_85_,- | 3 | A1-A2 | 9 | 10 |
|  |  | Panama | 1,9,12:l,v:1,5 | 3 | A1-A4 | 1 | 6-9 |
|  |  | Zaiman | 9,12:l,v:e,n,x | 1 | A1-A2 | 4 | 6 |
|  |  | Goettingen | 9,12:l,v:e,n,z_15_ | 1 | A1-A2 | 6 | 1 |
|  |  |  |  | 1 | A1-A4 | 1 | 4 |
|  |  | 9,12:l,v:- |  | 1 | A1-A4 | 1 | 6 |
|  |  | Itami | 9,12:l,z_13_:1,5 | 1 | A1-A2 | 3 | 10 |
|  |  | Napoli | 1,9,12:l,z_13_:e,n,x | 2 | A1-A2 | 13 | 18 |
|  |  |  |  | 1 | A1-A4 | 4 | 18 |
|  |  | Javiana | 1,9,12:l,z_28_:1,5 | 4^1^ | A1-A3 | 4-6 | 4-12 |
|  |  |  |  |  |  |  |  |
|  |  | Gallinarum | 1,9,12:-:- | 14^1^ | A1-A2 | 2-3 | 4-11 |
|  |  | **Group O:3,10 (E1)** |  |  |  |  |  |
|  |  | Monophasic variant | 3,10:e,h:- | 1 | A1-A2 | 64 | 5 |
|  |  | Anatum | 3,10{15}{15,34}:e,h:1,6:z_64_ | 4 | A1-A2 | 2-8 | 17-25 |
|  |  | Weltevreden | 3,10{15}:r:z_6_ | 2^1^ | A1-A2 | 22-31 | 26-34 |
|  |  | Lexington | 3,10{15}{15,34}: z_10_:1,5 | 1 | A1-A2 | 21 | 35 |
|  |  | **Group O:1,3,19 (E4)** |  |  |  |  |  |
|  |  | Senftenberg | 1,3,19:g,[s],t:- | 8 | A1-A2 | 38-50 | 16-50 |
|  |  | **Group O:11 (F)** |  |  |  |  |  |
|  |  | Epinay | 11:a:l,z_13_,z_28_ | 1 | A1-A2 | 12 | 44 |
|  |  | Maracaibo | 11:l,v:1,5 | 1 | A1-A4 | 1 | 5 |
|  |  | Rubislaw | 11:r:e,n,x | 1 | A1-A4 | 1 | 1 |
|  |  | **Group O:13 (G)** |  |  |  |  |  |
|  |  | Mississippi | 1,13,23:b:1,5 | 1 | A1-A2 | 13 | 2 |
|  |  | Grumpensis | 1,13,23:d:1,7 | 1 | A1-A2 | 15 | 2 |
|  |  | Poona | 1,13,22:z:1,6 | 2 | A1-A4 | 27-34 | 5 |
|  |  | Worthington | 1,13,23:z:l,w | 12 | A1-A3 | 27 | 16-22 |
|  |  | **Group O:6,14 (H)** |  |  |  |  |  |
|  |  | Carrau | 6,14,[24]:y:1,7 | 1 | A1-A5 | 7 | 10 |
|  |  | Madelia | 1,6,14,25:y:1,7 | 1 | A1-A5 | 8 | 12 |
|  |  | **Group O:16 (I)** |  |  |  |  |  |
|  |  | Gaminara | 16 :d :1,7 | 1 | A1-A2 | 26 | 22 |
|  |  | **Group O:18 (K)** |  |  |  |  |  |
|  |  | Cerro | 6,14,18:z_4_,z_23_:[1,5] | 1 | A1-A2 | 32 | 25 |
|  |  | **Group O:28 (M)** |  |  |  |  |  |
|  |  | Doorn | 28:i:1,2 | 1 | A1-A2 | 40 | 26 |
|  |  | Pomona | 28:y:1,7 | 2 | A1-A4 | 1 | 10-11 |
|  |  | **Group O:30 (N)** |  |  |  |  |  |
|  |  | Overvecht | 30:a:1,2 | 1 | A1-A2 | 7 | 14 |
|  |  | Urbana | 30:b:e,n,x | 2 | A1-A4 | 15 | 3 |
|  |  | **Group O:35 (O)** |  |  |  |  |  |
|  |  | Adelaide | 35:f,g:- | 1 | A1-A2 | 12 | 2 |
|  |  | **Group O:40 (R)** |  |  |  |  |  |
|  |  | Johannesburg | 1,40:b:e,n,x | 1 | A1-A4 | 15 | 3 |
|  |  | **Group O:42 (T)** |  |  |  |  |  |
|  |  | Portedeslilas | 1,42:l,v:1,6,7 | 1 | A1-A4 | 14 | 1 |
|  |  | **Group O:44 (V)** |  |  |  |  |  |
|  |  | Gamaba | 1,44:g,m,[s]:[1,6] | 1 | A1-A2 | 4 | 24 |
|  |  | Niarembe | 44:a:l,w | 1 | A1-A2 | 20 | 11 |
|  |  | **Group O:54** |  |  |  |  |  |
|  |  | Tonev | 21,54:b:e,n,x | 1 | A1-A2 | 14 | 16 |
|  |  | **Group O:67** |  |  |  |  |  |
|  |  | Crossness | 67:r:1,2 | 1 | A1-A2 | 18 | 18 |
|  | *salamae* | 6,7:l,w:1,5,7 |  | 1 | A1-A2 | 40 | 4 |
|  |  | 11:l,z_28_:enx |  | 1 | A1-A2 | 11 | 10 |
|  |  | 57:z_42_:1,6:z_53_ |  | 1 | A1-A2 | 17 | 9 |
|  |  | 58:l,z_13_,z_28_:z_6_ |  | 1 | A1-A2 | 37 | 14 |
|  | *arizonae* | 17:z_29_:- |  | 1 | A1-A3 | 1 | 0 |
|  |  | 53:g,z_51_:- |  | 1 | A1-A3 | 1 | 0 |
|  |  | 56:z_4_,z_23_:- |  | 1 | A1-A3 | 1 | 0 |
|  |  | 62:z_4_,z_23_:- |  | 2^1^ | A1-A3 | 1 | 0 |
|  | *diarizonae* | 38:z_10_:z_53_ |  | 1 | A1-A2 | 12 | 1 |
|  |  | 61:k:1,5,7 |  | 14 | A1-A2 | 3-22 | 1 |
|  |  | 61:l,v:1,5,7 |  | 1^1^ | A1-A2 | 30 | 1 |
|  | *houtenae* | 6,7:z_4_,z_24_:- |  | 1 | A1-A2 | 22 | 0 |
|  |  | 1,40:z_4_,z_24_:- |  | 1 | A1-A6 | 0 | 4 |
|  |  | 44:a:- |  | 1 | A1-A6 | 0 | 4 |
|  |  | 48:g,z_51_:- |  | 1 | A1-A2 | 14 | 0 |
|  | *indica* | 11:b:1,7 |  | 1 | A1-A2 | 8 | 4 |
|  |  | 6,7:z_41_:1,7 |  | 1 | A1-A3 | 1 | 16 |
| *S. bongori* |  | 60:z_41_:- |  | 1 | A1-A2 | 26 | 13 |
|  |  | 48:z_35_:- |  | 1 | A1-A2 | 2 | 11 |
|  |  | 66:z_35_:- |  | 1 | A1-A2 | 12 | 10 |
|  |  | 66:z_41_:- |  | 1^1^ | A1-A2 | 20 | 17 |

^1^ including one available genome sequence

^2^ including two available genome sequences

^3^ including seven available genome sequences
