# Supplementary material for: CRISPR Typing and Subtyping for Improved Laboratory Surveillance of Salmonella Infections
Source: PLoS One. 2012 May 18;7(5):e36995. doi: 10.1371/journal.pone.0036995 (PMC3356390; doi:10.1371/journal.pone.0036995)
Supplement: Table S5 — Comparison of CRISPR2 spacer content with the population structure of S. enterica serotype Newport as assessed by MLST. (DOC) [file pone.0036995.s007.doc]

**Table S5**. Comparison of CRISPR2 spacer content with the population structure of *S. enterica* serotype Newport as assessed by MLST

| **Lineage** | **Strain/isolate** | **MLST** | **CRISPR2 spacer content^2^** |
| --- | --- | --- | --- |
|  |  |  |  |
| **Newport-I** | 00-4093 | ST156 | SB0-SB32-SB2-SB3-SB4-SB5-SB6-SB7-SB8-SB8-SB10-SB11-SB17-HB21-HB22-NB28-NB29-NB30-NB31-NB18-SB19-NB19-NB20-NB21-NB22-NB23-NB24-NB25-NB26-NB27 |
|  | 01-2174 | ST156 | SB0-SB32-SB2-SB3-SB4-SB5-SB6-SB7-SB8-SB8-SB10-SB11-SB17-HB21-HB22-NB28-NB29-NB30-NB31-NB18-SB19-NB19-NB20-NB21-NB22-NB23-NB24-NB25-NB26-NB27 |
|  | 00-973 | ST166 | SB0-SB32-SB2-SB3-SB4-SB5-SB6-SB7-SB8-SB8-SB10-SB11-SB17-HB21-HB22-NB28-NB29-NB30-NB31-NB18-SB19-NB19-NB20-NB21-NB22-NB23-NB24-NB25-NB26-NB27 |
|  | 04-2487 | ST166 | SB0-SB32-SB2-SB3-SB4-SB5-SB6-SB7-SB8-SB10-SB10-SB11-SB17-HB21-HB22-NB28-NB29-NB30-NB31-NB18-SB19-NB19-NB20-NB21-NB22-NB23-NB24-NB25-NB26-NB27 |
|  | 39/64 | ST166 | SB0-SB32-SB2-SB3-SB4-SB5-SB6-SB7-SB8-SB10-SB11-SB17-HB21-HB22-NB28-NB29-NB30-NB31-NB18-SB19-NB19-NB20-NB21-NB22-NB23-NB24-NB25-NB26-NB27 |
|  |  |  |  |
| **Newport-II** | 10/66 | ST45 | ParAB0-InfB1-NB1-NB2-NB3-KotB2-NB41-NB4-NB5-NB6-NB7-NB8-NB9-NB10-NB11-NB12-NB13-NB14-NB15-NB16-NB17 |
|  | 00-4165 | ST45 | ParAB0-InfB1-NB1-NB2-NB3-NB4-NB5-NB6-NB7-NB8-NB9-NB10-NB11-NB12-NB13-NB14-NB15-NB16-NB17 |
|  | 02-7891 | ST45 | ParAB0-InfB1-NB1-NB2-NB3-NB4-NB5-NB6-NB7-NB8-NB9-NB10-NB11-NB12-NB13-NB14-NB15-NB16-NB17 |
|  | 04-9597 | ST45 | ParAB0-InfB1-NB1-NB2-NB3-NB4-NB5-NB6-NB7-NB8-NB9-NB10-NB11-NB12-NB13-NB14-NB15-NB16-NB17 |
|  | SL254^1^ | ST45 | ParAB0-InfB1-NB1-NB2-NB3-NB4-NB5-NB6-NB7-NB8-NB9-NB10-NB11-NB12-NB13-NB14-NB15-NB16-NB17 |
|  | 01-2010 | ND | ParAB0-InfB1-NB1-NB2-NB3-NB4-NB5-NB6-NB7-NB8-NB9-NB10-NB11-NB12-NB13-NB14-NB15-NB16-NB17 |
|  | 03-3224 | ND | ParAB0-InfB1-NB1-NB2-NB3-NB4-NB5-NB6-NB7-NB8-NB9-NB10-NB11-NB12-NB13-NB14-NB15-NB16-NB17 |
|  | 10/56 | ST46 | ParAB0-InfB1-NB1-NB2-KotB10-NB3-KotB2-NB41-NB32-NB33-KotB5-NB44-NB12-NB13-NB14-NB15-NB16-NB17 |
|  | 50K | ST31 | ParAB0-InfB1-NB1-NB5-NB6-NB7-NB8-NB9-NB10-NB32-NB33-KotB5-KotB6-NB11-NB12-NB13-NB14-NB15-NB16-NB17 |
|  | 04-1198 | ST31 | ParAB0-InfB1-NB1-NB5-NB6-KotB6-NB11-NB13-NB14-NB15-NB16-NB17 |
|  | 50/3 | ST31 | ParAB0-InfB1-NB1-NB5-NB6-NB33-KotB5-KotB6-NB11-NB12-NB13-NB14-NB15-NB16-NB17 |
|  | 2/58 | ST211 | ParAB0-InfB1-NB1-NB2-NB3-KotB2-NB41-NB43-NB12-NB13-NB14-NB15-NB16-NB17 |
|  |  |  |  |
| **Newport-III** | 05-0815 | ST118 | SB0-SB32-SB1-HB17-NB34-NB35-SB17-HB20-NB40-Mba6 |
|  | 4/51 | ST118 | SB0-SB32-SB1-HB17-NB34-SB17-HB21-SB34-0-NB30-NB31-NB29-NB18-NB37 |
|  | 03-8748 | ST118 | SB0-SB32-SB1-NB34-NB35-SB17-HB20-HB21-NB36-SB34-SB18-0-NB30-NB31-NB29-NB18-Mba6 |
|  | SL317^1^ | ST5 | SB0-SB32-SB1-HB17-NB34-NB35-SB17var1-HB20-HB21-NB36-SB18-0-NB30-NB31-NB29-NB18-NB37-NB38-Mba6 |

**^1^**Genome

**^2^**Due to space constraints, the spacer names NewpB, STMB and HadB are abbreviated to NB, SB and HB, respectively; 0 = no spacer between two contiguous DRs.
