# Supplementary material for: CRISPR Typing and Subtyping for Improved Laboratory Surveillance of Salmonella Infections
Source: PLoS One. 2012 May 18;7(5):e36995. doi: 10.1371/journal.pone.0036995 (PMC3356390; doi:10.1371/journal.pone.0036995)
Supplement: Table S6 — CRISPR2 spacer content in various O:9 and O:2 serotypes. (DOC) [file pone.0036995.s008.doc]

**Table S6**. CRISPR2 spacer content in various O:9 and O:2 serotypes

| **Serotype** | **Antigenic formula** | **Biotype** | **MLST** | **No. of isolates** | **CRISPR2 spacer content** |
| --- | --- | --- | --- | --- | --- |
| Enteritidis | 9,12:g,m:- |  | ST11 group^1^ |  |  |
|  |  |  |  | 2 | EntB0-EntB1-EntB10-EntB22-EntB23-EntB7-EntB9 |
|  |  |  |  | 1 | EntB0-EntB1-EntB2-EntB10-EntB3-EntB4-EntB5-EntB6-EntB7-EntB8-EntB9 |
|  |  |  |  | 1 | EntB0-EntB1-EntB2-EntB3-EntB4 |
|  |  |  |  | 1 | EntB0-EntB1-EntB2-EntB3-EntB4-EntB5-EntB6 |
|  |  |  |  | 51 | EntB0-EntB1-EntB2-EntB3-EntB4-EntB5-EntB6-EntB7-EntB8-EntB8-EntB9 |
|  |  |  |  | 102 | EntB0-EntB1-EntB2-EntB3-EntB4-EntB5-EntB6-EntB7-EntB8-EntB9 |
|  |  |  |  | 5^2^ | EntB0-EntB1-EntB2-IndB1 |
|  |  |  |  | 2 | EntB0-EntB1-EntB2-IndB1-EntB10-EntB3-EntB4-EntB5-EntB6-EntB7-EntB8-EntB9var1 |
|  |  |  |  | 1 | EntB0-EntB1-EntB2-IndB1-EntB10-EntB3-EntB4-EntB7-EntB8-EntB9 |
|  |  |  |  | 2 | EntB0-EntB1-EntB2-IndB1-EntB10-EntB3-EntB8-EntB9 |
|  |  |  |  | 2 | EntB0-EntB1-EntB2-IndB1-EntB6-EntB7-EntB8-EntB9 |
|  |  |  |  | 1 | EntB10-EntB3-EntB4-EntB5-EntB6-EntB7 |
|  |  |  |  | 1 | EntB4-EntB5-EntB6-EntB7-EntB8-EntB8-EntB9 |
|  |  |  | Other STs |  |  |
|  |  |  | ST180 | 1 | EntB0-EntB1-EntB2-IndB1-EntB11-EntB17-EntB21-EntB20 |
|  |  |  | ST180 | 1 | EntB0-EntB1-EntB2-IndB1-EntB12-EntB14-EntB18-EntB19-EntB21-EntB20 |
|  |  |  | ST180 | 1 | EntB0-EntB1-EntB2-IndB1-EntB24-EntB11-EntB12-EntB13-EntB14-EntB15-EntB16-EntB17-EntB18-EntB19-EntB21-EntB20 |
|  |  |  | ST6 | 1 | EntB39-EntB40-EntB41-EntB42-EntB43-EntB46-EntB47-EntB51-EntB52-EntB52-EntB54-EntB55-EntB56-EntB57-//-EntB45^3^ |
|  |  |  | ST77 | 1 | ParBB1-ParBB24-ParBB2-EntB25-EntB26-EntB27-StpB28-EntB28-EntB29-EntB30-EntB31-EntB32-MonB51-EntB33-//-EntB38^3^ |
|  | 9,12:g,m,p:- |  | ST74 | 1 | EntB0-EntB1-EntB2-IndB1-EntB11-EntB12-EntB13-EntB14-EntB15-EntB16-EntB17-EntB18-EntB19-EntB20 |
|  | 9,12:g,m,p:- |  | ST74 | 1 | EntB0-EntB1-EntB2-IndB1-EntB11-EntB12-EntB13-EntB14-EntB15-EntB16-EntB17-EntB18-EntB19-EntB21-EntB20 |
|  | 9,12:g,m:1,7 |  | ST746 | 1 | AgoB1-AgoB2-WorB1-WorB2-IstB7-EntB71-EntB72-EntB73-EntB74-Kot26var1-EntB75-EntB76-EntB77 |
|  |  |  |  |  |  |
| Blegdam | 9,12:g,m,q:- |  | ST739 (ST11 SLV) | 1 | EntB0-EntB1-EntB2-IndB1-EntB10-EntB3-EntB8-EntB9 |
|  |  |  |  |  |  |
| Rosenberg | 9,12:g,z85:- |  | ST11 | 3 | EntB0-EntB1-EntB2-EntB3-EntB4-EntB5-EntB5-EntB7-EntB8-EntB9 |
|  |  |  |  |  |  |
| Dublin | 9,12:g,p:- |  | ST10 | 3 | EntB0-EntB1-EntB1-EntB7-EntB8 |
|  |  |  | ST10 | 1 | EntB0-EntB1-EntB7-EntB8 |
|  |  |  | ST73 | 2 | EntB0-EntB1-EntB1-EntB7-EntB8 |
| Gallinarum | 9,12:-:- |  |  |  |  |
|  |  | Gallinarum | ST78 | 1 | EntB0-EntB1-GallB1-GallB2-EntB3-EntB4-EntB5-EntB6-EntB7-EntB8-EntB9 |
|  |  | Gallinarum | ST78 | 3 | EntB0-EntB2-GallB1-GallB2-EntB3-EntB4-EntB5-EntB7-EntB8-EntB9 |
|  |  | Gallinarum | ST78 | 3 | GallB1-GallB2-GallB2-EntB3-EntB4-EntB5-EntB7-EntB8-EntB9 |
|  |  | Pullorum | ST92 | 3 | EntB0-GallB1-GallB2-EntB3-EntB8-EntB9 |
|  |  | Pullorum | ST747 (ST92 SLV) | 1 | EntB0-GallB1-GallB2-EntB3-EntB8-EntB9 |
|  |  | Pullorum | ST92 | 1 | EntB0-GallB1-GallB2-EntB8-EntB9var2 |
|  |  | Duisburg^4^ | ST762 | 2 | EntB0-EntB4-EntB8-EntB9 |
|  |  |  |  |  |  |
| Nitra | 2,12:g,m:- |  | ST11 | 2 | EntB0-EntB1-EntB2-EntB3-EntB4-EntB5-EntB6-EntB7-EntB8-EntB9 |
|  |  |  | ST11 | 1 | EntB0-EntB1-EntB2-IndB1-EntB10-EntB3-EntB8-EntB9 |
|  |  |  |  |  |  |
| Kiel | 2,12:g,p:- |  | ST10 | 1 | EntB0-EntB1-EntB7 |
|  |  |  | ST10 | 1 | EntB0-EntB1-EntB7-EntB7-EntB8 |
|  |  |  | ST10 | 1 | EntB0-EntB1-EntB7-EntB8 |

^1^ST (sequence type) 11 group consists of ST11 and its single-locus variants (SLV)

^2^Includes the 5 ST136 “Danysz” strains used as rodenticides

^3^EntB57-//-EntB45, 18 unique spacers are located between EntB57 and EntB45; EntB33-//-EntB38, 4 unique spacers are located between EntB33 and EntB38  (see Table S2)

^4^Serotype Gallinarum biovar Duisburg is different from serotype Duisburg
