# Supplementary material for: CRISPR Typing and Subtyping for Improved Laboratory Surveillance of Salmonella Infections
Source: PLoS One. 2012 May 18;7(5):e36995. doi: 10.1371/journal.pone.0036995 (PMC3356390; doi:10.1371/journal.pone.0036995)
Supplement: Table S8 — Epidemiological concordance of CRISPR spacer content for separate outbreaks due to serotype Enteritidis. (DOC) [file pone.0036995.s010.doc]

**Table S8**. Epidemiological concordance of CRISPR spacer content for separate outbreaks due to serotype Enteritidis

| **Outbreak no.** | **Year** | **Isolate** | **Outbreak-related** | **Source** | **CRISPR1 spacer content** | **CRISPR2 spacer content** | **XbaI PFGE/PT** |
| --- | --- | --- | --- | --- | --- | --- | --- |
| 1 | 2001 | 01-4146 | Yes | Human | Ent1-Ent2-Ent3-Ent4-Ent5-Ent6-Ent7-Ent9-Ent8 | EntB0-EntB1-EntB2-EntB3-EntB4-EntB5-EntB6-EntB7-EntB8-EntB9 | XENT-1/PT4 |
| 1 | 2001 | 01-4197 | Yes | Human | Ent1-Ent2-Ent3-Ent4-Ent5-Ent6-Ent7-Ent9-Ent8 | EntB0-EntB1-EntB2-EntB3-EntB4-EntB5-EntB6-EntB7-EntB8-EntB9 | XENT-1/PT4 |
| 1 | 2001 | 01-4224 | Yes | Human | Ent1-Ent2-Ent3-Ent4-Ent5-Ent6-Ent7-Ent9-Ent8 | EntB0-EntB1-EntB2-EntB3-EntB4-EntB5-EntB6-EntB7-EntB8-EntB9 | XENT-1/PT4 |
| 1 | 2001 | 01- 4874 | Yes | Sausage | Ent1-Ent2-Ent3-Ent4-Ent5-Ent6-Ent7-Ent9-Ent8 | EntB0-EntB1-EntB2-EntB3-EntB4-EntB5-EntB6-EntB7-EntB8-EntB9 | XENT-1/PT4 |
| 1 | 2001 | 01 -3703 | No | Human | Ent1-Ent2-Ent3-Ent4-Ent5-Ent6-Ent7-Ent8 | EntB0-EntB1-EntB2-EntB3-EntB4-EntB5-EntB6-EntB7-EntB8-EntB9 | XENT-1/PT4 |
| 2 | 2002 | 02 -9386 | Yes | Human | Ent1-Ent2-Ent3-Ent4-Ent5-Ent7-Ent8 | EntB0-EntB1-EntB2-EntB3-EntB4-EntB5-EntB6-EntB7-EntB8-EntB9 | XENT-1/PT4 |
| 2 | 2002 | 02 -9439 | Yes | Human | Ent1-Ent2-Ent3-Ent4-Ent5-Ent7-Ent8 | EntB0-EntB1-EntB2-EntB3-EntB4-EntB5-EntB6-EntB7-EntB8-EntB9 | XENT-1/PT4 |
| 2 | 2002 | 02 -9440 | Yes | Human | Ent1-Ent2-Ent3-Ent4-Ent5-Ent7-Ent8 | EntB0-EntB1-EntB2-EntB3-EntB4-EntB5-EntB6-EntB7-EntB8-EntB9 | XENT-1/PT4 |
| 2 | 2002 | 02-9451 | Yes | Human | Ent1-Ent2-Ent3-Ent4-Ent5-Ent7-Ent8 | EntB0-EntB1-EntB2-EntB3-EntB4-EntB5-EntB6-EntB7-EntB8-EntB9 | XENT-1/PT4 |
| 2 | 2002 | 02 -9453 | Yes | Environment hen layer building C | Ent1-Ent2-Ent3-Ent4-Ent5-Ent7-Ent8 | EntB0-EntB1-EntB2-EntB3-EntB4-EntB5-EntB6-EntB7-EntB8-EntB9 | XENT-1/PT35 |
| 2 | 2002 | 02 -9455 | Yes | Environment hen layer building B | Ent1-Ent2-Ent3-Ent4-Ent5-Ent7-Ent8 | EntB0-EntB1-EntB2-EntB3-EntB4-EntB5-EntB6-EntB7-EntB8-EntB9 | XENT-1/PT35 |
| 2 | 2002 | 02 -9456 | Yes | Stools hen building B | Ent1-Ent2-Ent3-Ent4-Ent5-Ent7-Ent8 | EntB0-EntB1-EntB2-EntB3-EntB4-EntB5-EntB6-EntB7-EntB8-EntB9 | XENT-1/PT6a |
| 2 | 2002 | 02 -9458 | Yes | Environment hen layer building B | Ent1-Ent2-Ent3-Ent4-Ent5-Ent7-Ent8 | EntB0-EntB1-EntB2-EntB3-EntB4-EntB5-EntB6-EntB7-EntB8-EntB9 | XENT-1/PT35 |
| 2 | 2002 | 02 -9459 | Yes | Stools hen building A | Ent1-Ent2-Ent3-Ent4-Ent5-Ent7-Ent8 | EntB0-EntB1-EntB2-EntB3-EntB4-EntB5-EntB6-EntB7-EntB8-EntB9 | XENT-1/PT4 |
| 3 | 2003 | 03 -3459 | Yes | Human | Ent1-Ent2var1-Ent3-Ent4-Ent5-Ent6-Ent7-Ent9-Ent8 | EntB0-EntB1-EntB2-EntB3-EntB4-EntB5-EntB6-EntB7-EntB8-EntB8-EntB9 | XENT-2/PT14b |
| 3 | 2003 | 03 -3460 | Yes | Human | Ent1-Ent2var1-Ent3-Ent4-Ent5-Ent6-Ent7-Ent9-Ent8 | EntB0-EntB1-EntB2-EntB3-EntB4-EntB5-EntB6-EntB7-EntB8-EntB8-EntB9 | XENT-2/PT14b |
| 3 | 2003 | 03- 3527 | Yes | Human | Ent1-Ent2var1-Ent3-Ent4-Ent5-Ent6-Ent7-Ent9-Ent8 | EntB0-EntB1-EntB2-EntB3-EntB4-EntB5-EntB6-EntB7-EntB8-EntB8-EntB9 | XENT-2/PT14b |
| 3 | 2003 | 03 -3697 | Yes | Human | Ent1-Ent2var1-Ent3-Ent4-Ent5-Ent6-Ent7-Ent9-Ent8 | EntB0-EntB1-EntB2-EntB3-EntB4-EntB5-EntB6-EntB7-EntB8-EntB8-EntB9 | XENT-2/PT14b |
| 3 | 2003 | 03 -3698 | Yes | Human | Ent1-Ent2var1-Ent3-Ent4-Ent5-Ent6-Ent7-Ent9-Ent8 | EntB0-EntB1-EntB2-EntB3-EntB4-EntB5-EntB6-EntB7-EntB8-EntB8-EntB9 | XENT-2/PT14b |
| 3 | 2003 | 03 -4022 | Yes | Human | Ent1-Ent2var1-Ent3-Ent4-Ent5-Ent6-Ent7-Ent9-Ent8 | EntB0-EntB1-EntB2-EntB3-EntB4-EntB5-EntB6-EntB7-EntB8-EntB8-EntB9 | XENT-2/PT14b |
| 3 | 2003 | 03 -4162 | Yes | Spring roll | Ent1-Ent2var1-Ent3-Ent4-Ent5-Ent6-Ent7-Ent9-Ent8 | EntB0-EntB1-EntB2-EntB3-EntB4-EntB5-EntB6-EntB7-EntB8-EntB8-EntB9 | XENT-2/PT14b |
| 3 | 2003 | 03- 4163 | Yes | Spring roll | Ent1-Ent2var1-Ent3-Ent4-Ent5-Ent6-Ent7-Ent9-Ent8 | EntB0-EntB1-EntB2-EntB3-EntB4-EntB5-EntB6-EntB7-EntB8-EntB8-EntB9 | XENT-2/PT14b |
| 3 | 2003 | 03 -4164 | Yes | Spring roll | Ent1-Ent2var1-Ent3-Ent4-Ent5-Ent6-Ent7-Ent9-Ent8 | EntB0-EntB1-EntB2-EntB3-EntB4-EntB5-EntB6-EntB7-EntB8-EntB8-EntB9 | XENT-2/PT14b |
| 3 | 2003 | 03- 4165 | Yes | Chicken meat | Ent1-Ent2var1-Ent3-Ent4-Ent5-Ent6-Ent7-Ent9-Ent8 | EntB0-EntB1-EntB2-EntB3-EntB4-EntB5-EntB6-EntB7-EntB8-EntB8-EntB9 | XENT-2/PT14b |
| 4 | 2001 | 01 -3970 | Yes | Human | Ent1-Ent2var1-Ent3-Ent4-Ent5-Ent6-Ent7-Ent9-Ent8 | EntB0-EntB1-EntB2-EntB3-EntB4-EntB5-EntB6-EntB7-EntB8-EntB8-EntB9 | XENT-2/PT8 |
| 4 | 2001 | 01 -4050 | Yes | Human | Ent1-Ent2var1-Ent3-Ent4-Ent5-Ent6-Ent7-Ent9-Ent8 | EntB0-EntB1-EntB2-EntB3-EntB4-EntB5-EntB6-EntB7-EntB8-EntB8-EntB9 | XENT-2/PT8 |
| 4 | 2001 | 01- 4118 | Yes | Human | Ent1-Ent2var1-Ent3-Ent4-Ent5-Ent6-Ent7-Ent9-Ent8 | EntB0-EntB1-EntB2-EntB3-EntB4-EntB5-EntB6-EntB7-EntB8-EntB8-EntB9 | XENT-2/PT8 |
| 4 | 2001 | 01 -5200 | Yes | Human | Ent1-Ent2var1-Ent3-Ent4-Ent5-Ent6-Ent7-Ent9-Ent8 | EntB0-EntB1-EntB2-EntB3-EntB4-EntB5-EntB6-EntB7-EntB8-EntB8-EntB9 | XENT-2/PT8 |
| 4 | 2001 | 01 -6091 | Yes | Human | Ent1-Ent2var1-Ent3-Ent4-Ent5-Ent6-Ent7-Ent9-Ent8 | EntB0-EntB1-EntB2-EntB3-EntB4-EntB5-EntB6-EntB7-EntB8-EntB8-EntB9 | XENT-2/PT8 |
| 4 | 2001 | 01-6649 | Yes | Human | Ent1-Ent2var1-Ent3-Ent4-Ent5-Ent6-Ent7-Ent9-Ent8 | EntB0-EntB1-EntB2-EntB3-EntB4-EntB5-EntB6-EntB7-EntB8-EntB8-EntB9 | XENT-2/PT8 |
| 4 | 2001 | 01 -6091 | Yes | Human | Ent1-Ent2var1-Ent3-Ent4-Ent5-Ent6-Ent7-Ent9-Ent8 | EntB0-EntB1-EntB2-EntB3-EntB4-EntB5-EntB6-EntB7-EntB8-EntB8-EntB9 | XENT-2/PT8 |
| 4 | 2001 | 01- 6649 | Yes | Raw milk cheese | Ent1-Ent2var1-Ent3-Ent4-Ent5-Ent6-Ent7-Ent9-Ent8 | EntB0-EntB1-EntB2-EntB3-EntB4-EntB5-EntB6-EntB7-EntB8-EntB8-EntB9 | XENT-2/PT8 |
