# Supplementary material for: CRISPR Typing and Subtyping for Improved Laboratory Surveillance of Salmonella Infections
Source: PLoS One. 2012 May 18;7(5):e36995. doi: 10.1371/journal.pone.0036995 (PMC3356390; doi:10.1371/journal.pone.0036995)
Supplement: Table S9 — Probe responses in the CRISPOL assay for triplicates of 5 individual strains or isolates. (DOC) [file pone.0036995.s011.doc]

**Table S9**. Probe responses in the CRISPOL assay for triplicates of 5 individual isolates or strains

| **Probe name**  **(bead no.)** | **Strain #LT2**  Median (MFI) ± SD^1^ | **Strain #SARA7**  Median (MFI) ± SD | **Isolate #02-7015**  Median (MFI) ± SD | **Isolate #07-1777**  Median (MFI) ± SD | **Isolate #81-831**  Median (MFI) ± SD |
| --- | --- | --- | --- | --- | --- |
| pBraB14 (29) | 67 ± 4 | 4401 ± 87 | 57 ± 4 | 59 ± 4 | 63 ± 3 |
| pSTMB35 (30) | 62 ± 3 | 3950 ± 96 | 59 ± 5 | 66 ± 8 | 78 ± 5 |
| pHadB20 (31) | 59 ± 6 | 1671 ± 39 | 1440 ± 46 | 1432 ± 13 | 60 ± 2 |
| pSTM01 (32) | 2074 ± 59 | 2492 ± 60 | 2228 ± 93 | 2074 ± 62 | 2203 ± 90 |
| pSTM02 (33) | 2606 ± 39 | 2757 ± 10 | 2455 ± 88 | 2543 ± 36 | 2484 ± 75 |
| pSTM03 (34) | 3156 ± 82 | 3758 ± 67 | 3568 ± 121 | 3304 ± 43 | 3519 ± 141 |
| pSTM03var1 (35) | 1801 ± 20 | 2247 ± 52 | 2064 ± 71 | 1835 ± 30 | 1936 ± 62 |
| pSTM04 (36) | 3508 ± 51 | 4225 ± 39 | 3927 ± 119 | 3798 ± 45 | 3839 ± 54 |
| pSTM05 (37) | 4556 ± 90 | 5121 ± 11 | 4746 ± 111 | 4668 ± 145 | 4743 ± 67 |
| pSTM06 (38) | 2682 ± 48 | 3021 ± 30 | 2574 ± 73 | 2612 ± 47 | 2665 ± 72 |
| pSTM07 (39) | 4092 ± 54 | 63 ± 5 | 4130 ± 67 | 4152 ± 194 | 4274 ± 33 |
| pSTM07var2 (40) | 2819 ± 19 | 65 ± 1 | 2895 ± 42 | 2796 ± 15 | 2874 ± 19 |
| pSTM08 (41) | 742 ± 10 | 57 ± 3 | 704 ± 27 | 704 ± 4 | 651 ± 16 |
| pSTM09 (42) | 3623 ± 49 | 64 ± 4 | 3828 ± 49 | 3722 ± 54 | 72 ± 8 |
| pSTM10 (43) | 3812 ± 11 | 87 ± 5 | 3513 ± 58 | 3768 ± 48 | 91 ± 3 |
| pSTM11 (44) | 4024 ± 50 | 62 ± 5 | 49 ± 2 | 4094 ± 31 | 65 ± 2 |
| pSTM12 (45) | 3370 ± 30 | 63 ± 5 | 3307 ± 129 | 2942 ± 48 | 71 ± 2 |
| pSTM12var1 (46) | 1213 ± 30 | 61 ± 5 | 1169 ± 37 | 944 ± 12 | 62 ± 5 |
| pSTM13 (47) | 3183 ± 55 | 60 ± 1 | 3321 ± 109 | 3233 ± 61 | 3572 ± 84 |
| pSTM14 (48) | 3906 ± 42 | 55 ± 4 | 3953 ± 146 | 4003 ± 49 | 4067 ± 116 |
| pSTM15 (49) | 2995 ± 46 | 3484 ± 30 | 3241 ± 101 | 2993 ± 83 | 3404 ± 12 |
| pSTM16 (50) | 3608 ± 96 | 69 ± 5 | 3830 ± 71 | 3609 ± 80 | 4914 ± 94 |
| pSTM17 (51) | 223 ± 64 | 75 ± 3 | 2332 ± 72 | 2028 ± 27 | 113 ± 16 |
| pSTM18 (52) | 3580 ± 35 | 2818 ± 25 | 3544 ± 43 | 3560 ± 120 | 82 ± 2 |
| pSTM19 (53) | 3146 (+/-53 | 3405 ± 41 | 3112 ± 115 | 3142 ± 88 | 3193 ± 108 |
| pSTM20 (54) | 2439 ± 28 | 3032 ± 71 | 2703 ± 79 | 2587 ± 23 | 2671 ± 43 |
| pSTM21 (55) | 2532 ± 30 | 70 ± 5 | 3423 ± 71 | 3525 ± 111 | 82 ± 12 |
| pSTM22 (56) | 3771 ± 36 | 67 ± 8 | 2313 ± 116 | 2098 ± 7 | 74 ± 2 |
| pSTM24 (57) | 57 ± 6 | 4666 ± 54 | 4313 ± 95 | 4215 ± 38 | 4221 ± 38 |
| pSTM25 (58) | 54 ± 1 | 4114 ± 17 | 3854 ± 56 | 3803 ± 70 | 3832 ± 55 |
| pSTM26 (59) | 53 ± 4 | 4465 ± 40 | 4368 ± 22 | 4192 ± 37 | 4274 ± 39 |
| pSTM27 (60) | 50 ± 3 | 3900 ± 101 | 3669 ± 118 | 3561 ± 61 | 3651 ± 54 |
| pSTM28 (61) | 53 ± 5 | 56 ± 1 | 3917 ± 148 | 4242 ± 60 | 66 ± 3 |
| pSTM29 (62) | 55 ± 6 | 64 ± 6 | 3107 ± 46 | 3155 ± 81 | 72 ± 6 |
| pSTM30 (63) | 73 ± 11 | 4739 ± 70 | 4325 ± 94 | 4232 ± 54 | 79 ± 6 |
| pSTM31 (64) | 58 ± 2 | 65 ± 1 | 3261 ± 124 | 3203 ± 10 | 71 ± 10 |
| pSTMB0 (65) | 3571 ± 68 | 4030 ± 13 | 3531 ± 147 | 3568 ± 69 | 3606 ± 131 |
| pSTMB01 (66) | 4891 ± 21 | 5369 ± 72 | 4944 ± 93 | 4879 ± 87 | 5084 ± 47 |
| pSTMB02 (67) | 3346 ± 24 | 3879 ± 9 | 57 ± 5 | 3480 ± 37 | 3498 ± 63 |
| pSTMB03 (68) | 2269 ± 32 | 2254 ± 32 | 52 ± 4 | 2154 ± 62 | 2061 ± 31 |
| pSTMB04 (69) | 4172 ± 41 | 4912 ± 17 | 59 ± 5 | 4263 ± 115 | 4405 ± 79 |
| pSTMB05 (70) | 4675 ± 46 | 5272 ± 66 | 56 ± 5 | 4733 ± 19 | 4682 ± 131 |
| pSTMB06 (71) | 2041 ± 15 | 1787 ± 8 | 62 ± 6 | 1816 ± 28 | 1645 ± 30 |
| pSTMB07 (72) | 4024 ± 113 | 4479 ± 18 | 58 ± 6 | 4057 ± 77 | 4040 ± 18 |
| pSTMB08 (73) | 3659 ± 53 | 4212 ± 32 | 64 ± 2 | 3693 ± 150 | 1810 ± 9 |
| pSTMB08var1 (74) | 987 ± 25 | 1179 ± 17 | 62 ± 3 | 947 ± 27 | 3873 ± 64 |
| pSTMB09 (75) | 1306 ± 15 | 1163 ± 16 | 62 ± 7 | 1133 ± 18 | 1050 ± 15 |
| pSTMB10 (76) | 2168 ± 16 | 2513 ± 41 | 60 ± 6 | 2166 ± 25 | 2157 ± 60 |
| pSTMB11 (77) | 1669 ± 29 | 2059 ± 17 | 1753 ± 49 | 1687 ± 27 | 1693 ± 30 |
| pSTMB12 (78) | 1829 ± 14 | 69 ± 4 | 62 ± 11 | 1856 ± 62 | 1928 ± 25 |
| pSTMB13 (79) | 4300 ± 74 | 67 ± 4 | 4334 ± 209 | 4201 ± 45 | 4611 ± 150 |
| pSTMB14 (80) | 2057 ± 46 | 58 ± 1 | 1978 ± 17 | 1949 ± 41 | 1810 ± 24 |
| pSTMB15 (81) | 3151 ± 32 | 67 ± 1 | 3283 ± 132 | 3197 ± 62 | 3263 ± 45 |
| pSTMB16 (82) | 4097 ± 45 | 76 ± 3 | 4285 ± 90 | 4323 ± 57 | 4365 ± 88 |
| pSTMB17 (83) | 2764 ± 40 | 99 ± 8 | 2700 ± 95 | 2780 ± 37 | 2675 ± 51 |
| pSTMB18 (84) | 3076 ± 69 | 3492 ± 82 | 3009 ± 140 | 2991 ± 139 | 3140 ± 45 |
| pSTMB19 (85) | 4120 ± 30 | 4173 ± 62 | 3968 ± 115 | 3987 ± 67 | 3968 ± 86 |
| pSTMB20 (86) | 3843 ± 24 | 4313 ± 61 | 71 ± 5 | 77 ± 4 | 4068 ± 75 |
| pSTMB21 (87) | 3184 ± 32 | 3629 ± 21 | 63 ± 5 | 66 ± 6 | 3307 ± 79 |
| pSTMB22 (88) | 4750 ± 136 | 5439 ± 40 | 71 ± 5 | 76 ± 8 | 5152 ± 96 |
| pSTMB23 (89) | 4650 ± 92 | 5413 ± 19 | 62 ± 7 | 62 ± 4 | 4930 ± 47 |
| pSTMB24 (90) | 3135 ± 68 | 3665 ± 51 | 64 ± 3 | 67 ± 2 | 3145 ± 118 |
| pSTMB25 (91) | 2077 ± 27 | 2080 ± 54 | 63 ± 1 | 67 ± 9 | 1872 ± 81 |
| pSTMB26 (92)^2^ | 3607 ± 70 | 659 ± 45 | 897 ± 153 | 639 ± 35 | 193 ± 15 |
| pSTMB27 (93) | 3310 ± 55 | 3548 ± 29 | 78 ± 2 | 94 ± 2 | 112 ± 8 |
| pSTMB28 (94) | 3083 ± 43 | 81 ± 4 | 77 ± 6 | 82 ± 4 | 95 ± 8 |
| pSTMB29 (95) | 3299 ± 54 | 90 ± 2 | 76 ± 5 | 84 ± 9 | 3053 ± 51 |
| pSTMB30 (96) | 2886 ± 10 | 80 ± 4 | 73 ± 6 | 74 ± 6 | 89 ± 3 |
| pSTMB31 (97) | 70 ± 5 | 2109 ± 19 | 78 ± 9 | 75 ± 1 | 97 ± 8 |
| pSTMB32 (98) | 3933 ± 54 | 4660 ± 81 | 4091 ± 93 | 4046 ± 36 | 4174 ± 111 |
| pSTMB33 (99) | 70 ± 4 | 2142 ± 16 | 1755 ± 39 | 1702 ± 13 | 1809 ± 40 |
| pSTMB34 (100) | 70 ± 5 | 3218 ± 59 | 2954 ± 63 | 2997 ± 50 | 83 ± 3 |

^1^MFI, median fluorescence intensity (raw data); SD, standard deviation

^2^There is a trimodal distribution with this probe. The strains or isolates **#**SARA7, **#**02-7015 and **#**07-1777 do not contain spacer STMB26 despite the stronger signal (which remains one third to one quarter that of truly positive isolates). This is due to the partial identity of pSTMB26 to spacer STMB34, which is found mostly in the emerging monophasic population. This has been taken into account by subtracting the MFI control strain **#**02-7015 from that of pSTMB26 in each experiment.
